# Supplementary figures and images for: Simultaneous TE Analysis of 19 Heliconiine Butterflies Yields Novel Insights into Rapid TE-Based Genome Diversification and Multiple SINE Births and Deaths
Source: Genome Biol Evol. 2019 Jun 19;11(8):2162–77. doi: 10.1093/gbe/evz125 (PMC6685494; doi:10.1093/gbe/evz125)

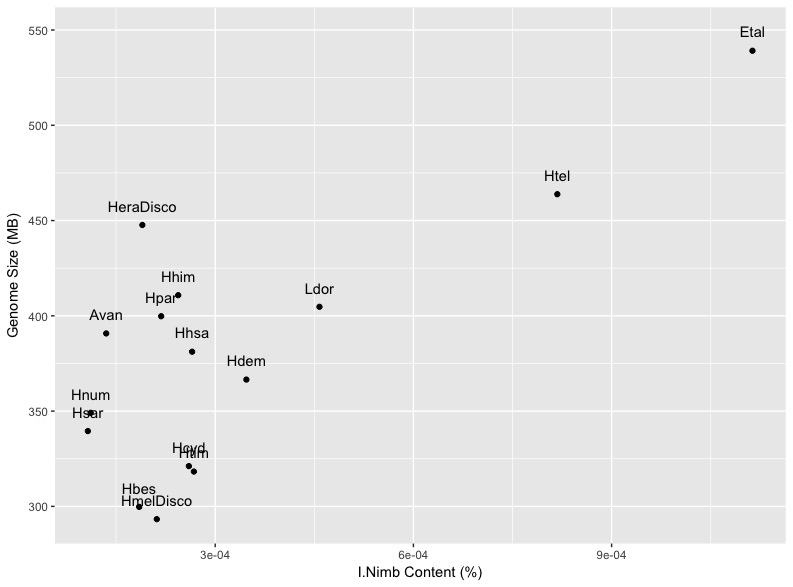

Supplement: evz125_Supplementary_Data [file evz125_supplementary_data.zip › Supplemental_Figure_10_-_I-Nimb_correlation.jpeg]

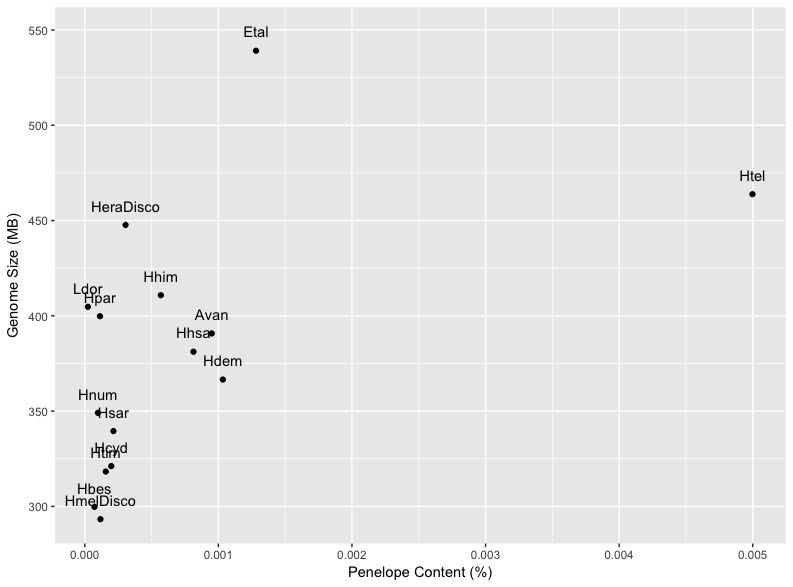

Supplement: evz125_Supplementary_Data [file evz125_supplementary_data.zip › Supplemental_Figure_11_-_Penelope_correlation.jpeg]

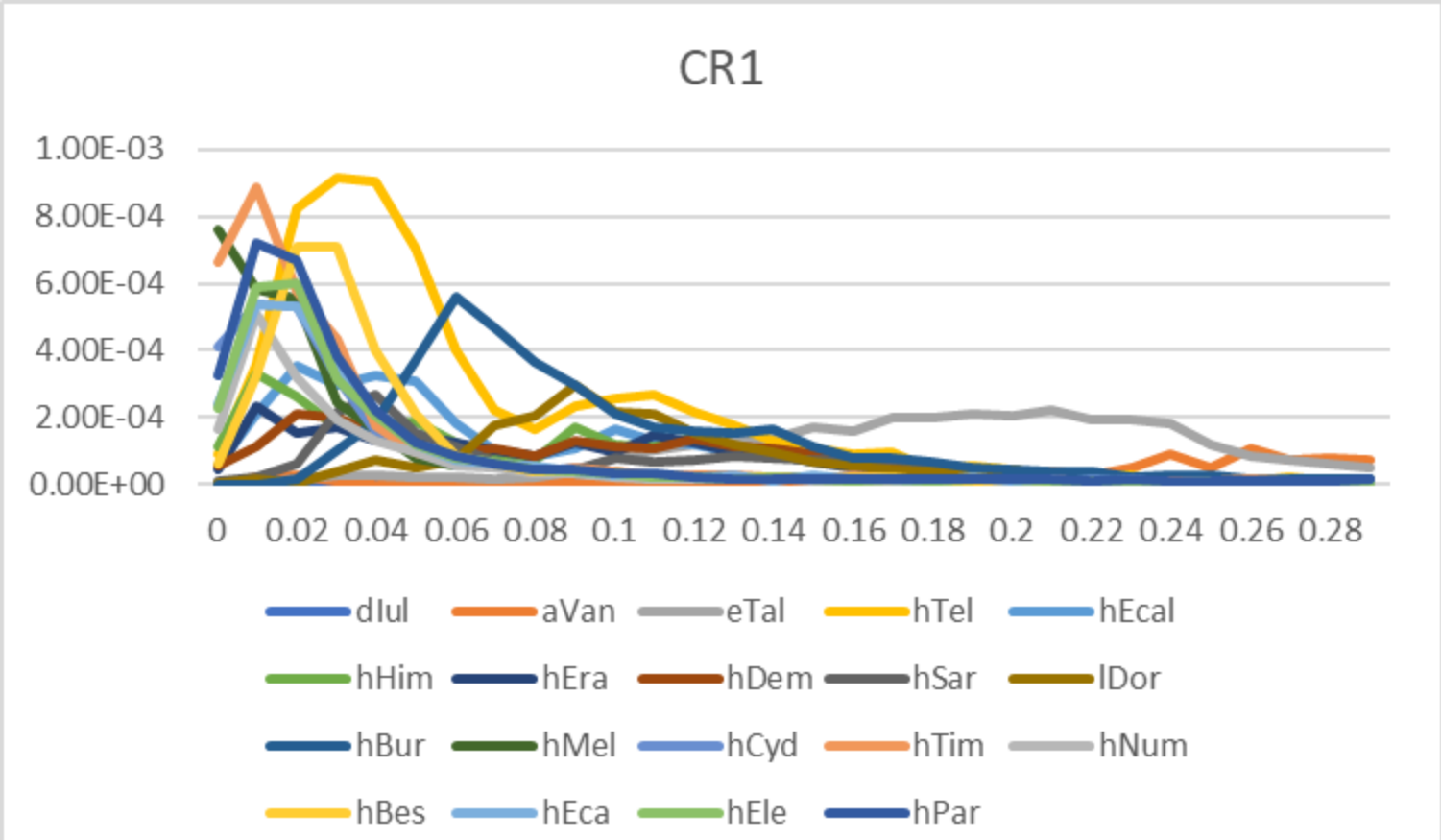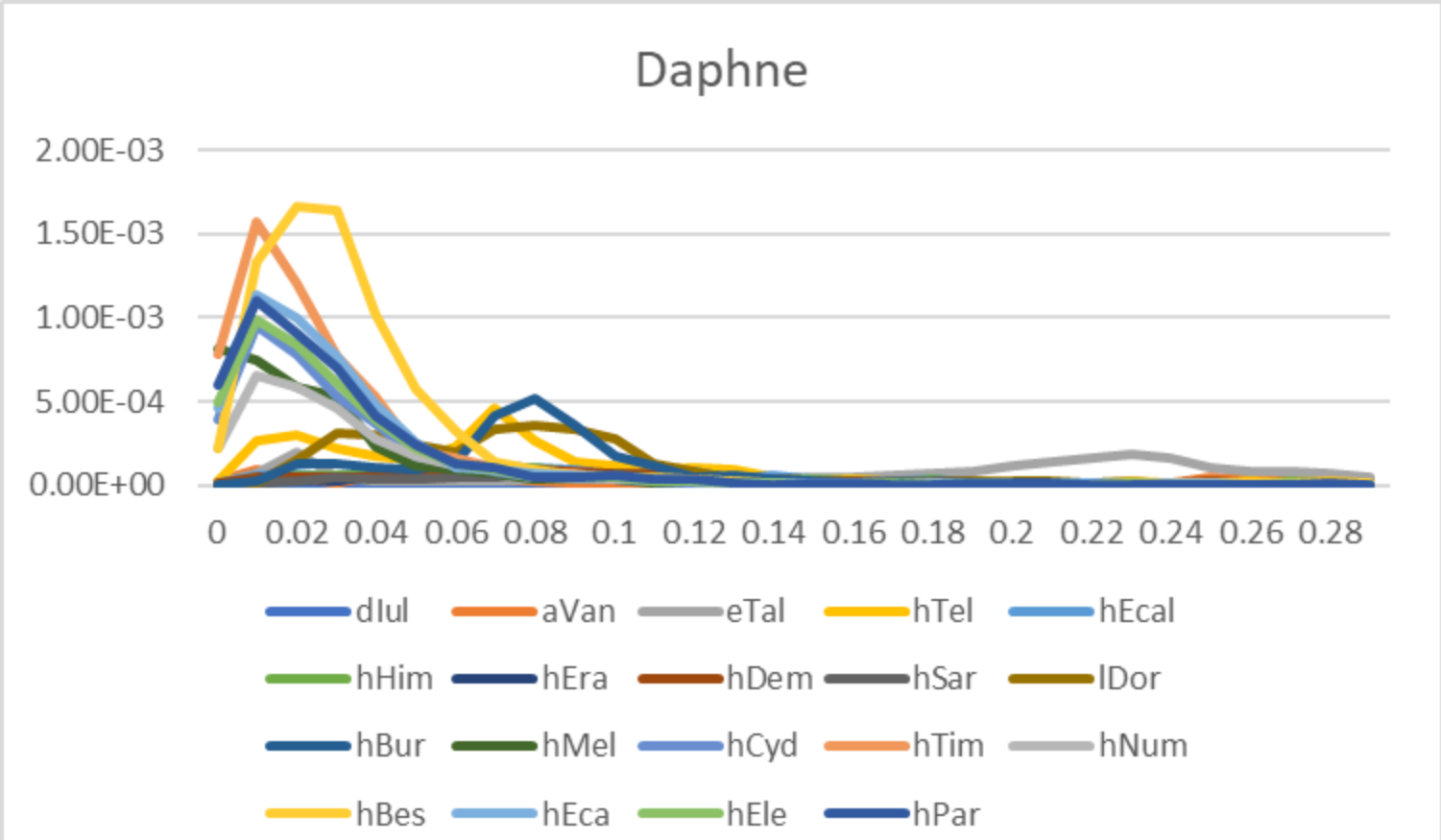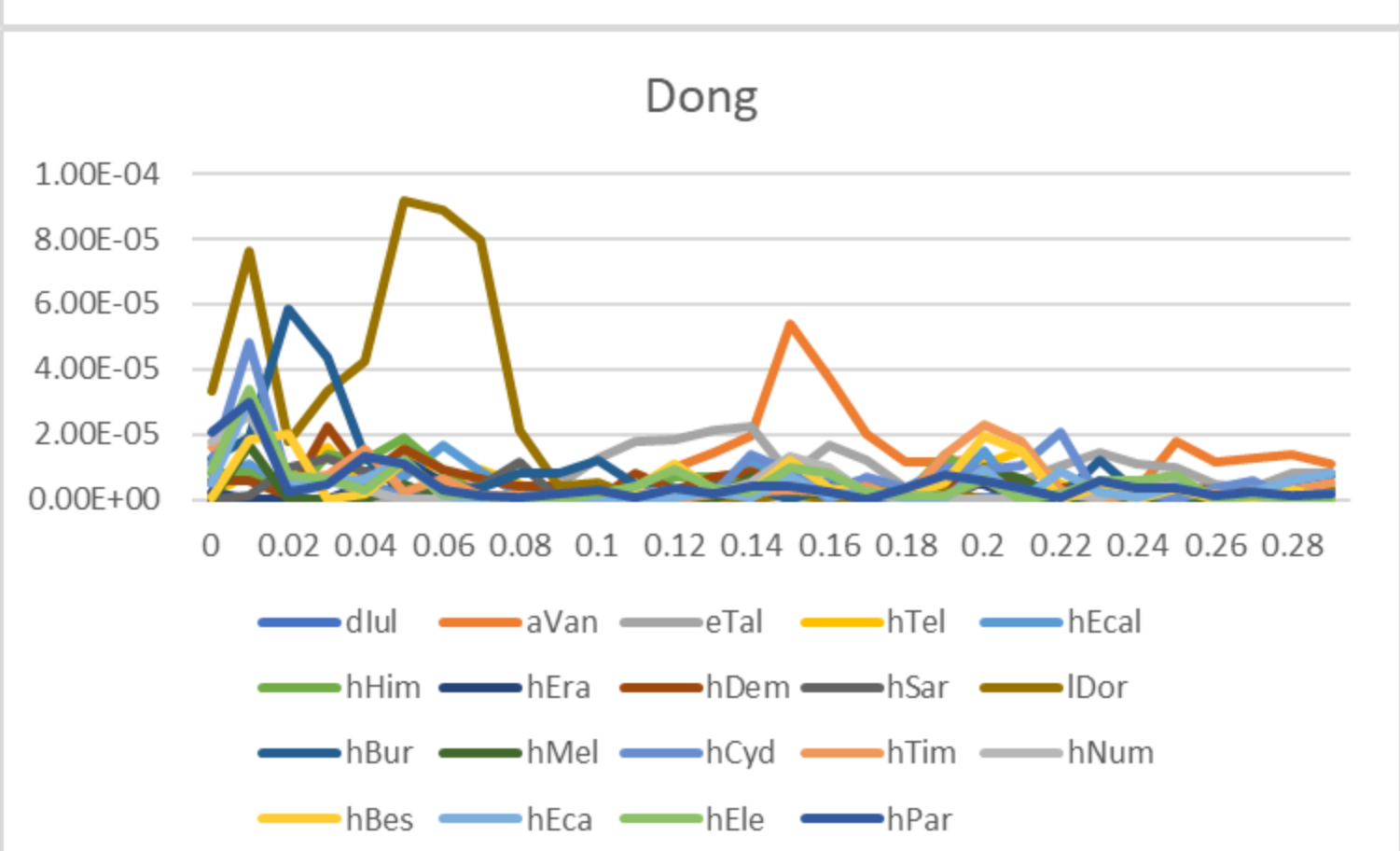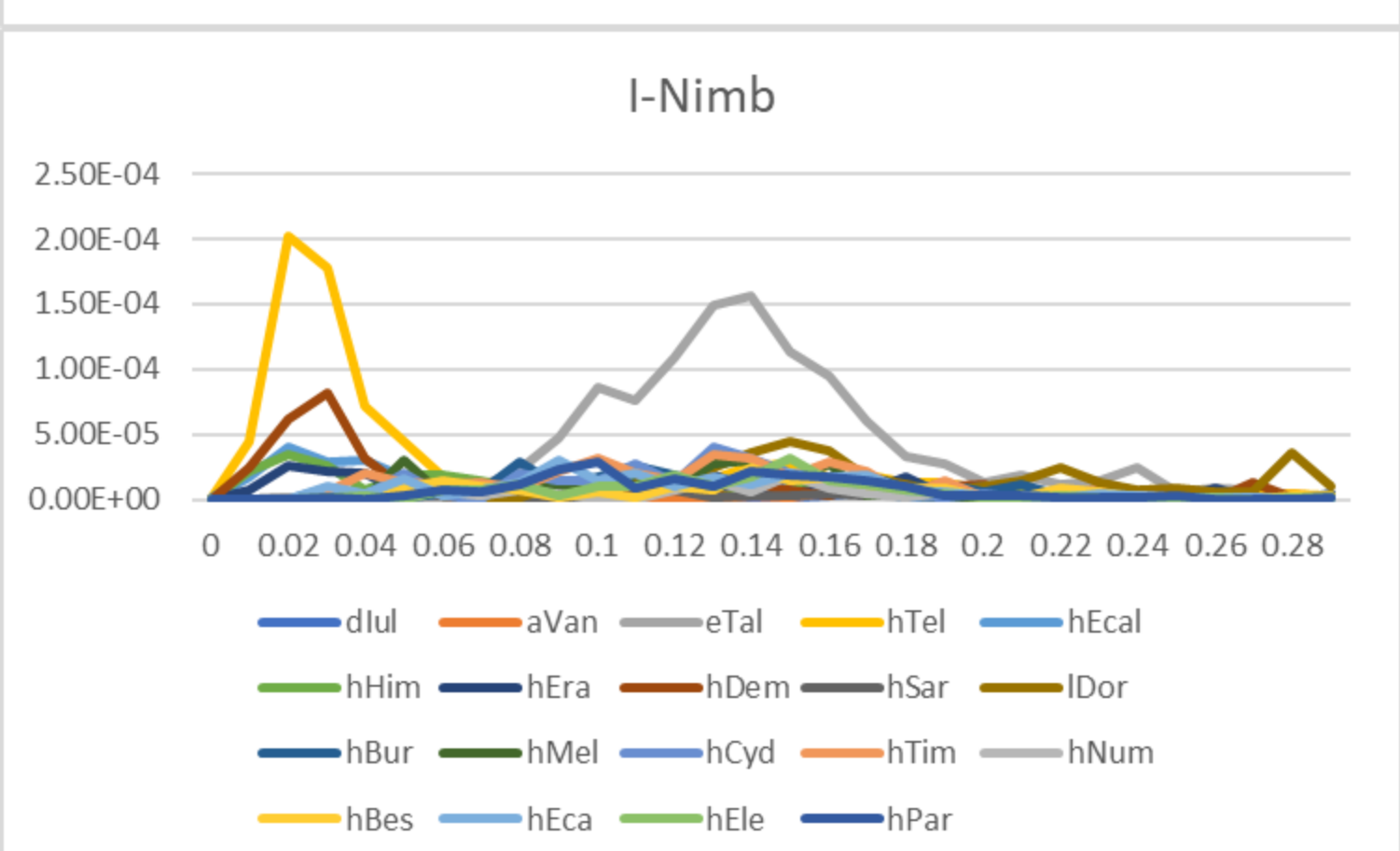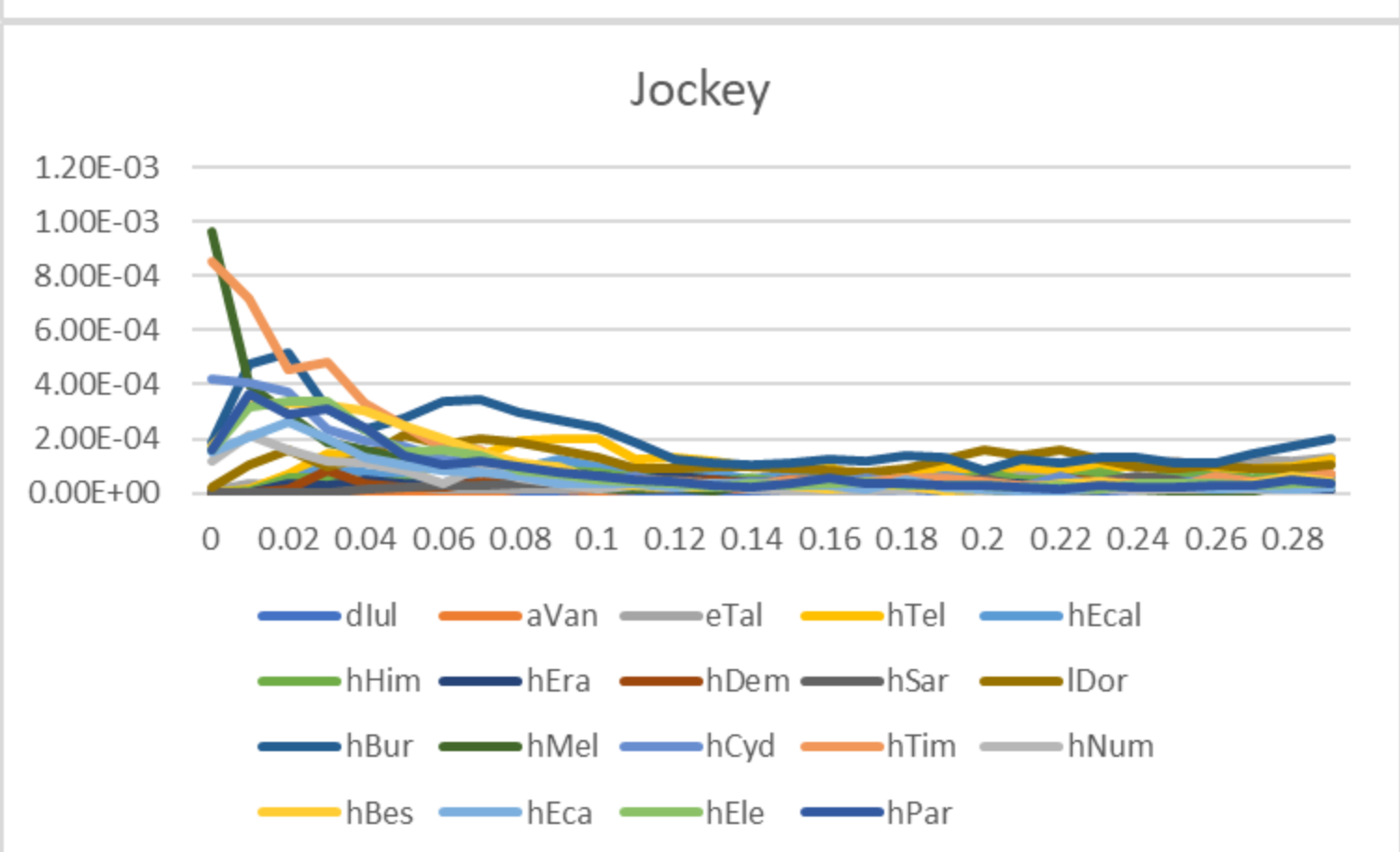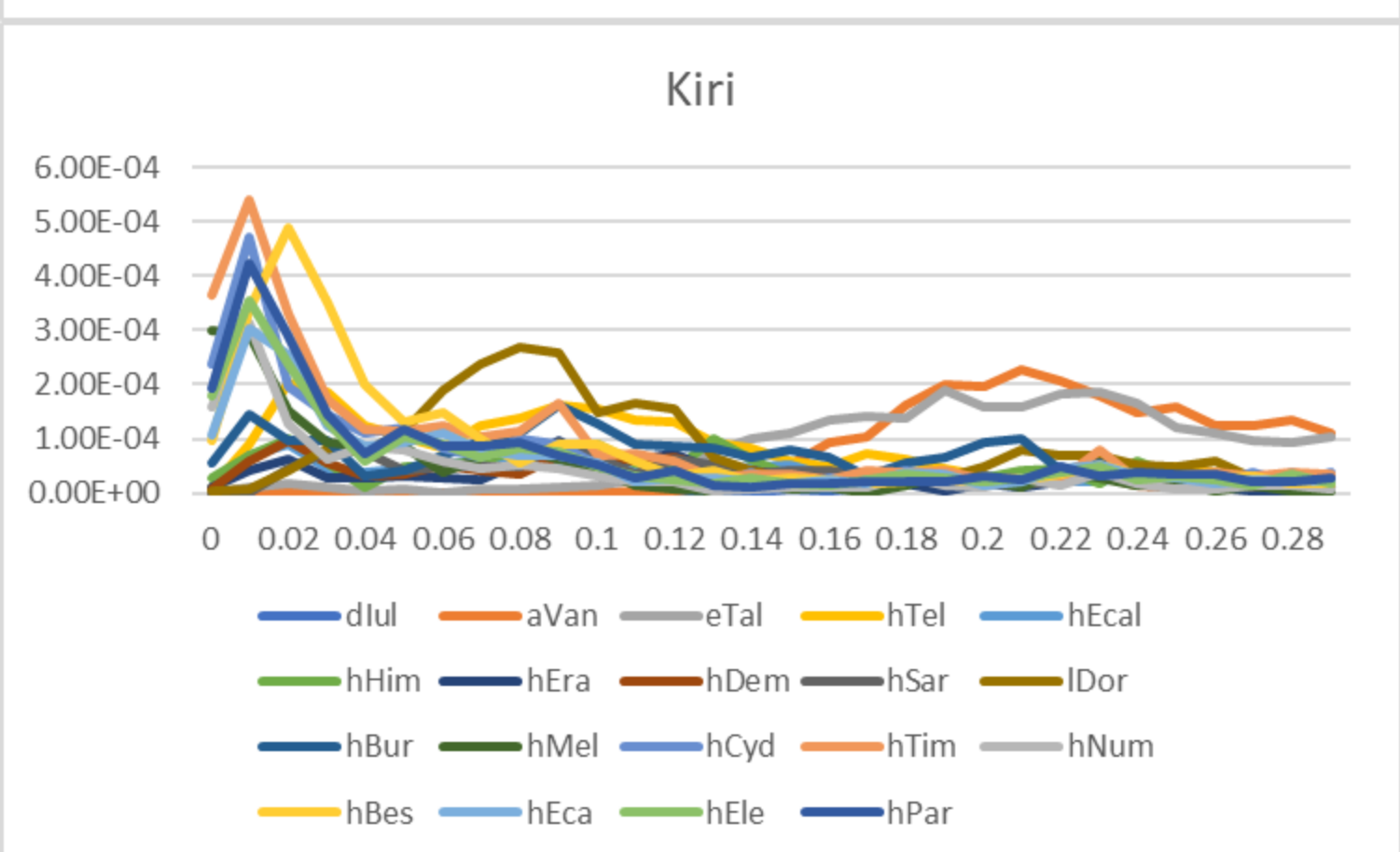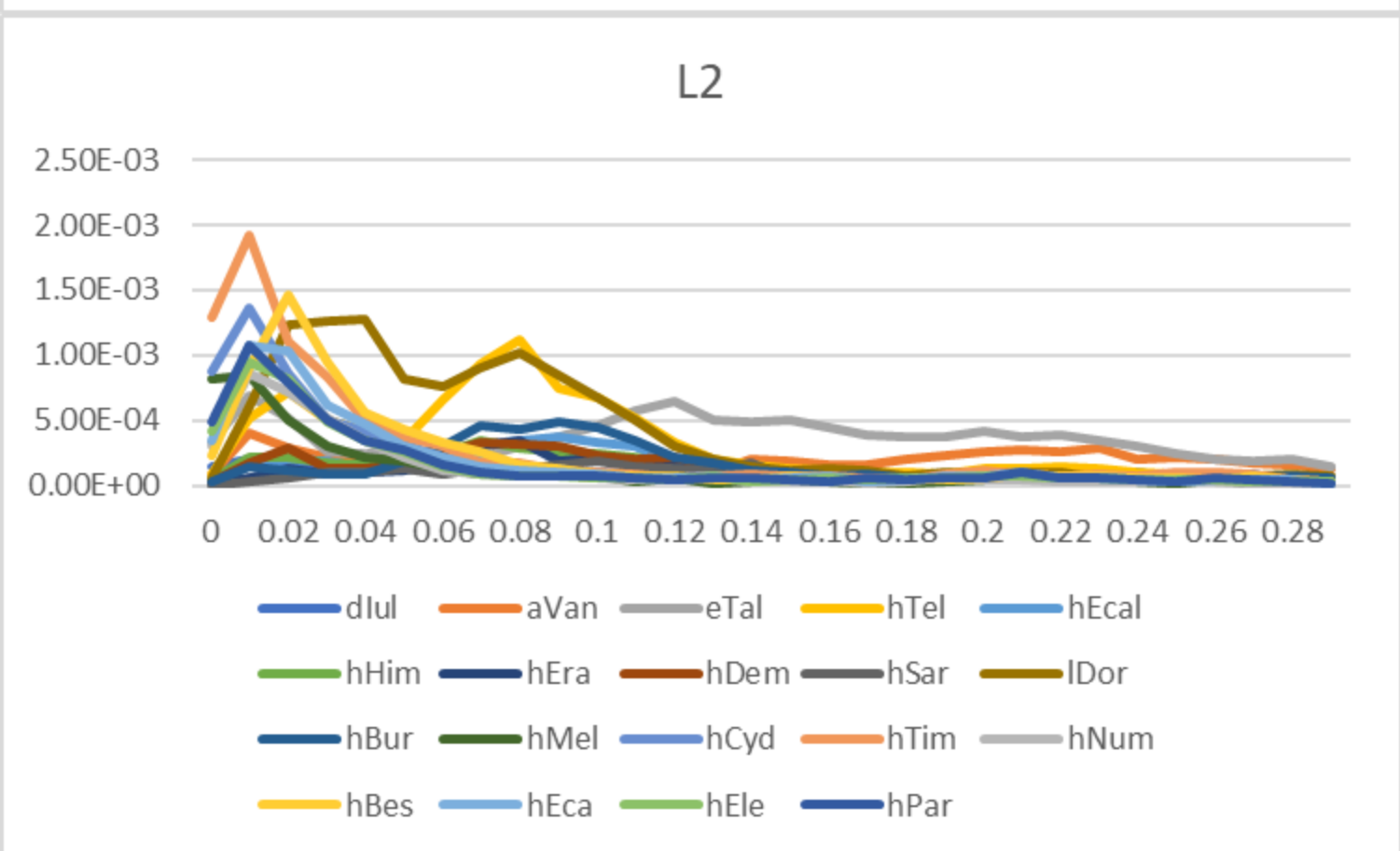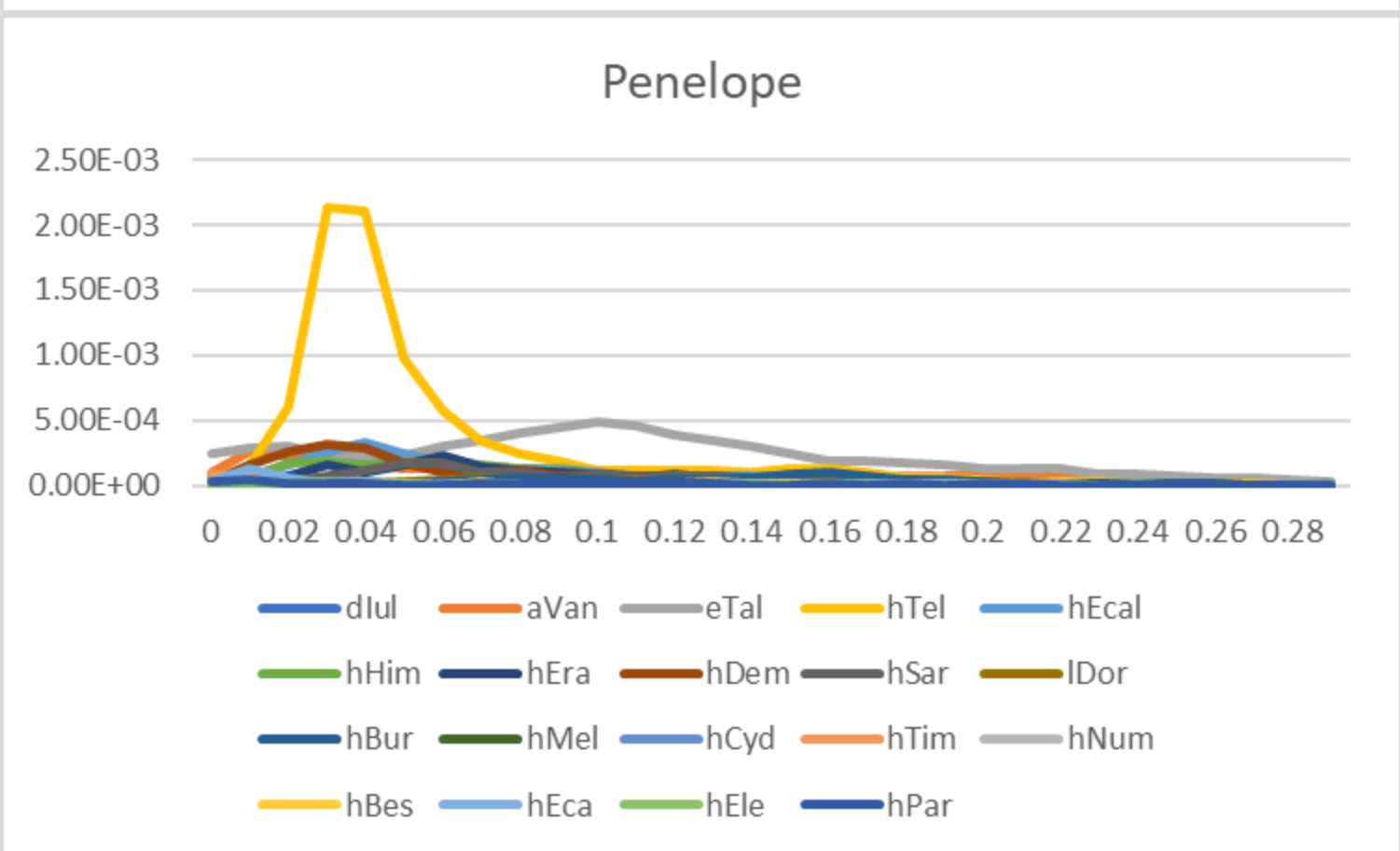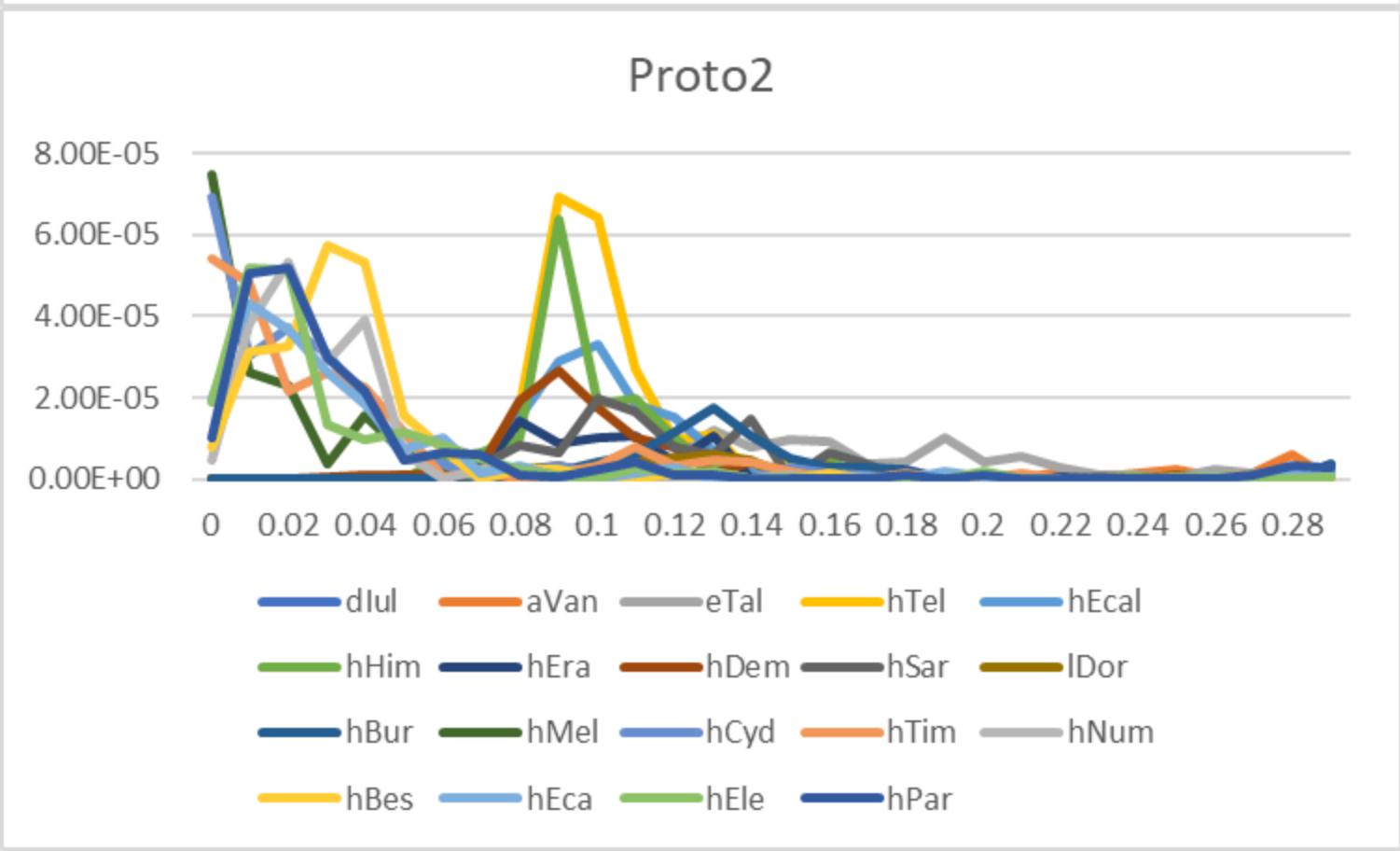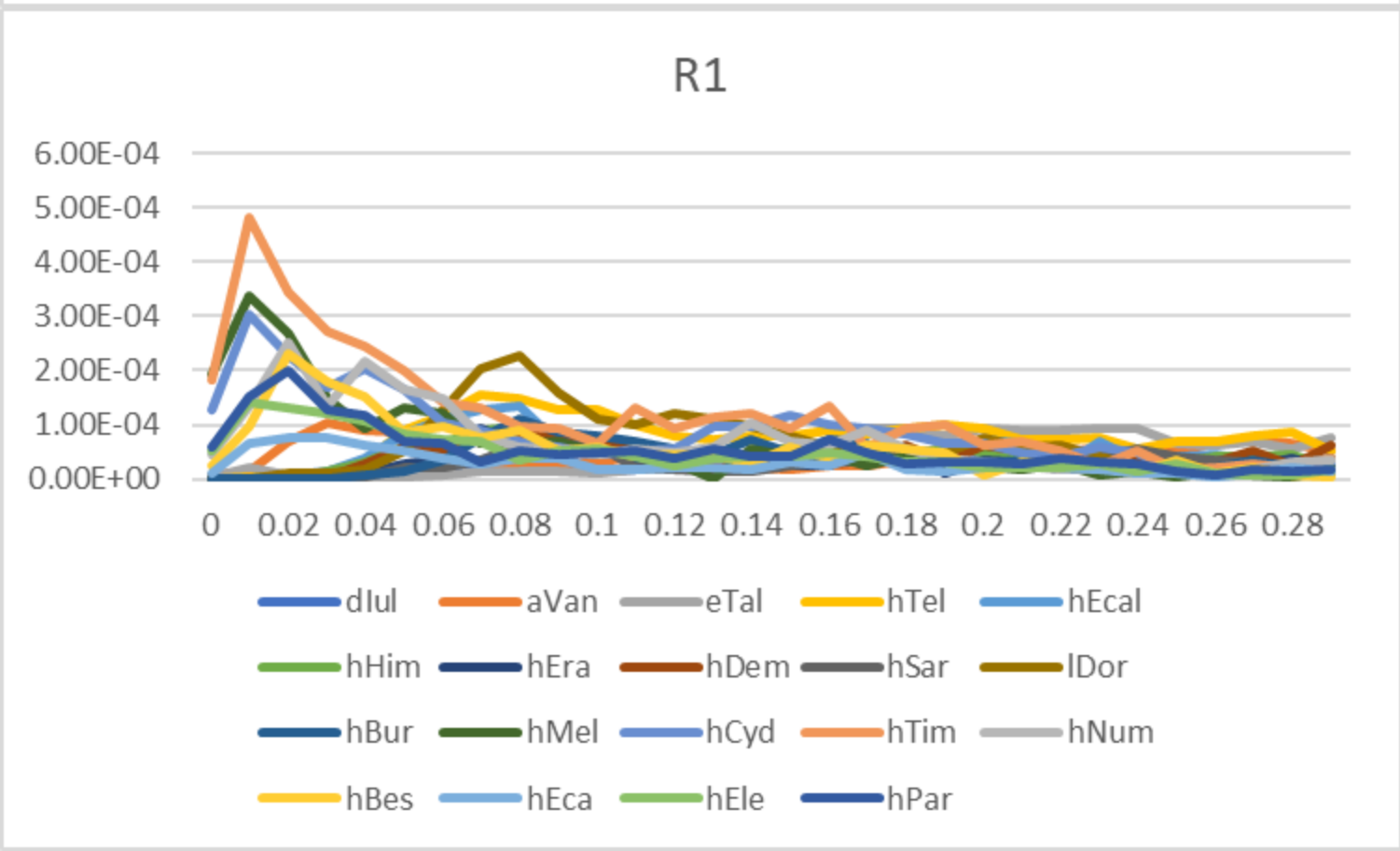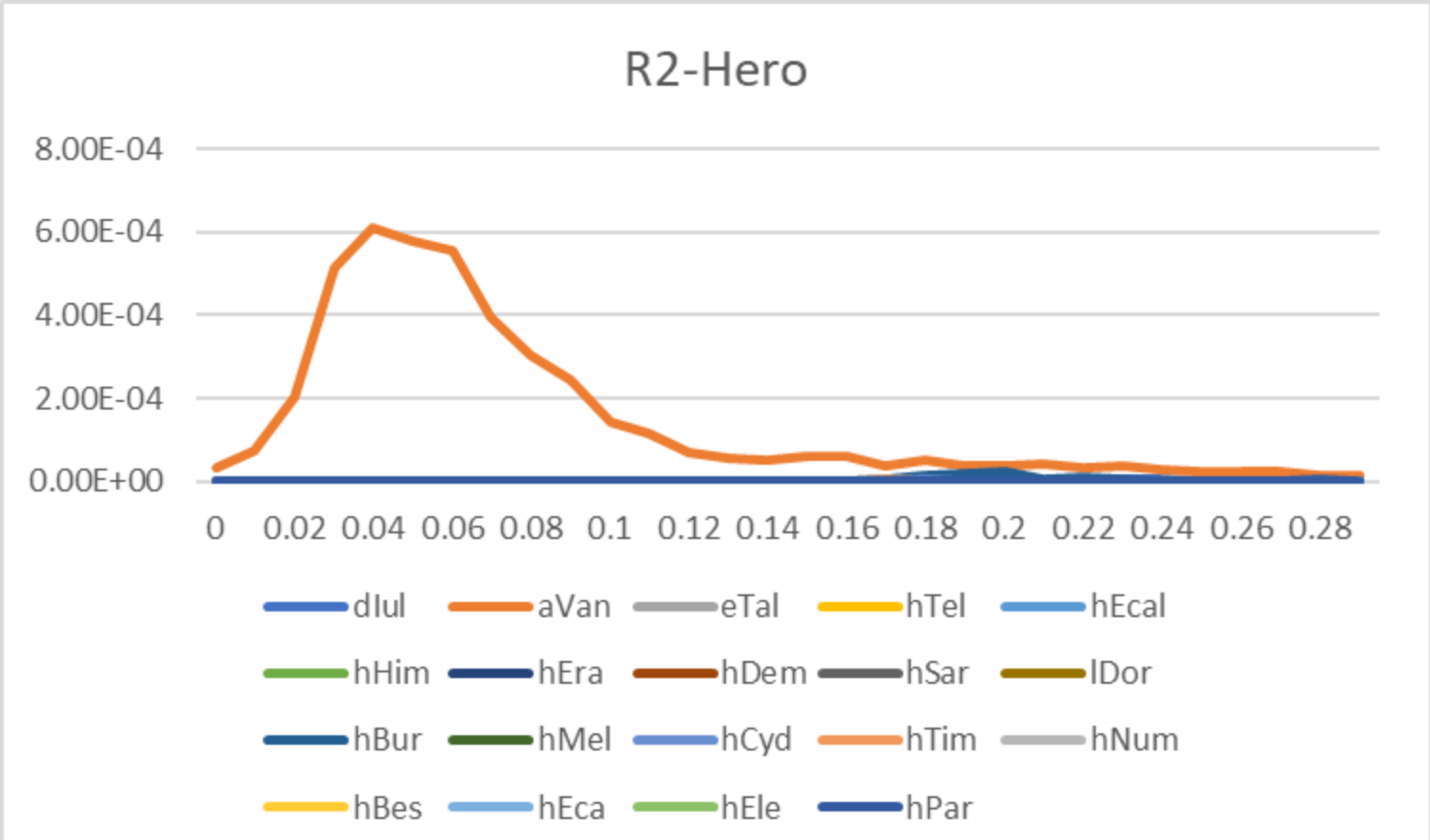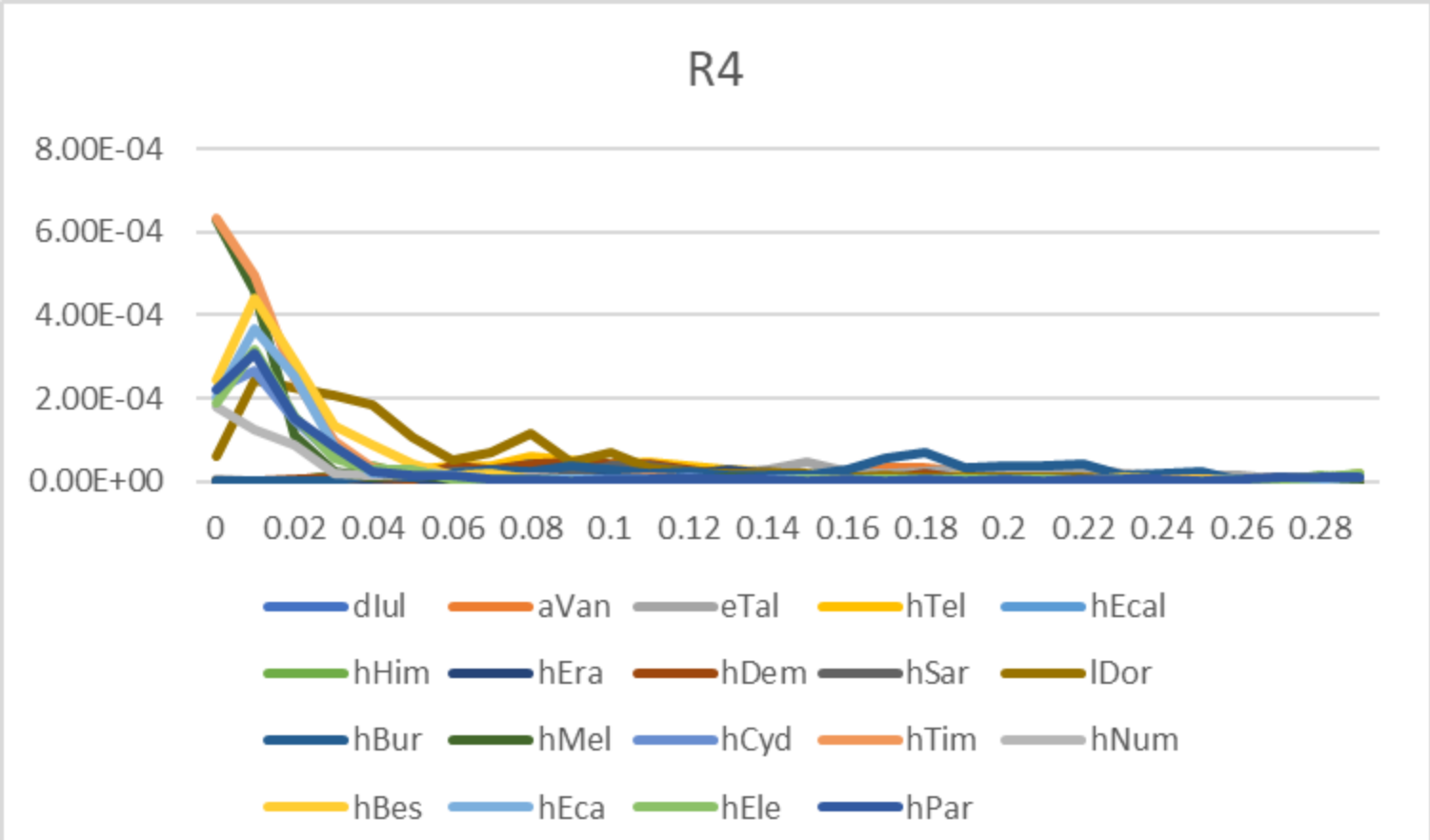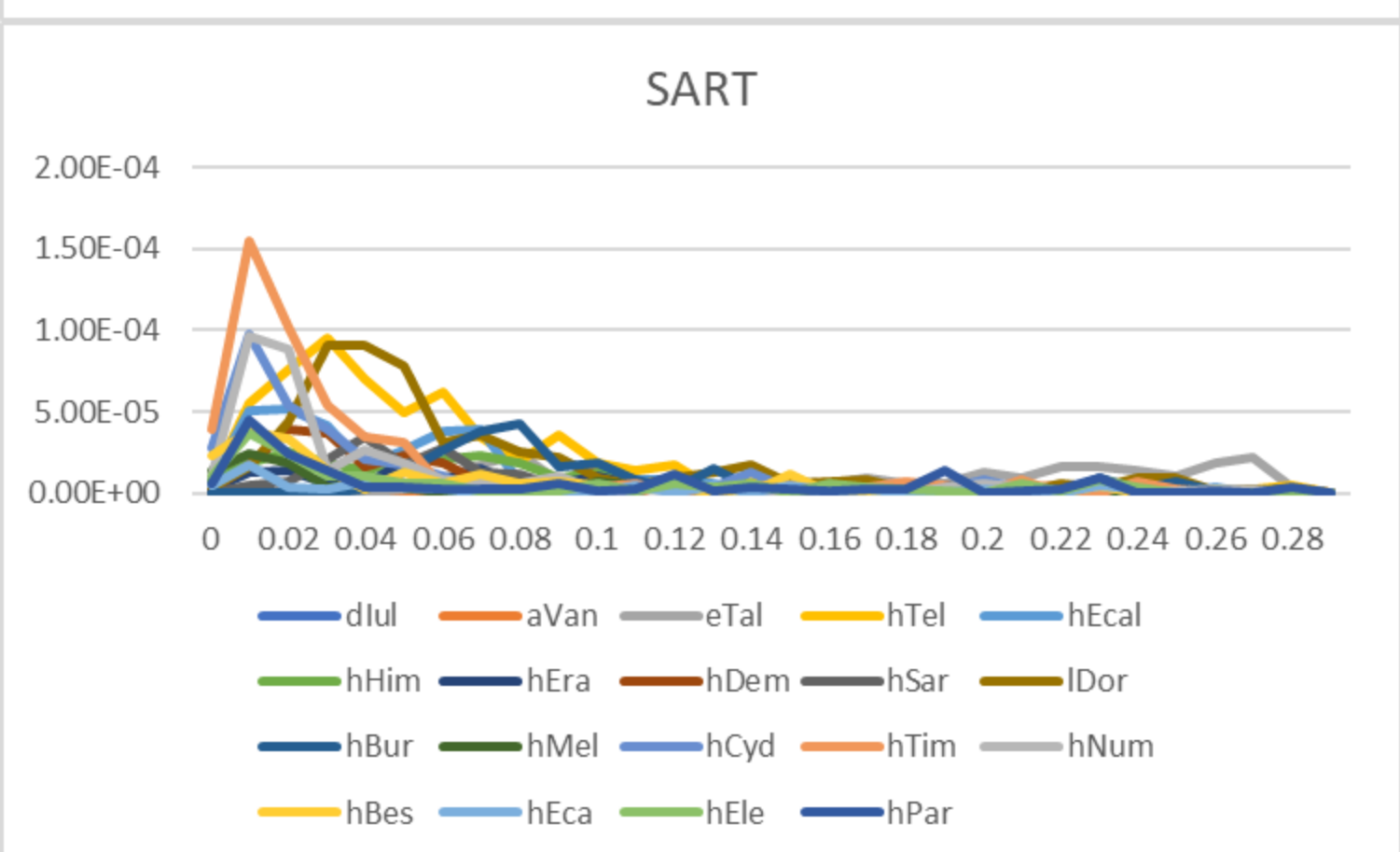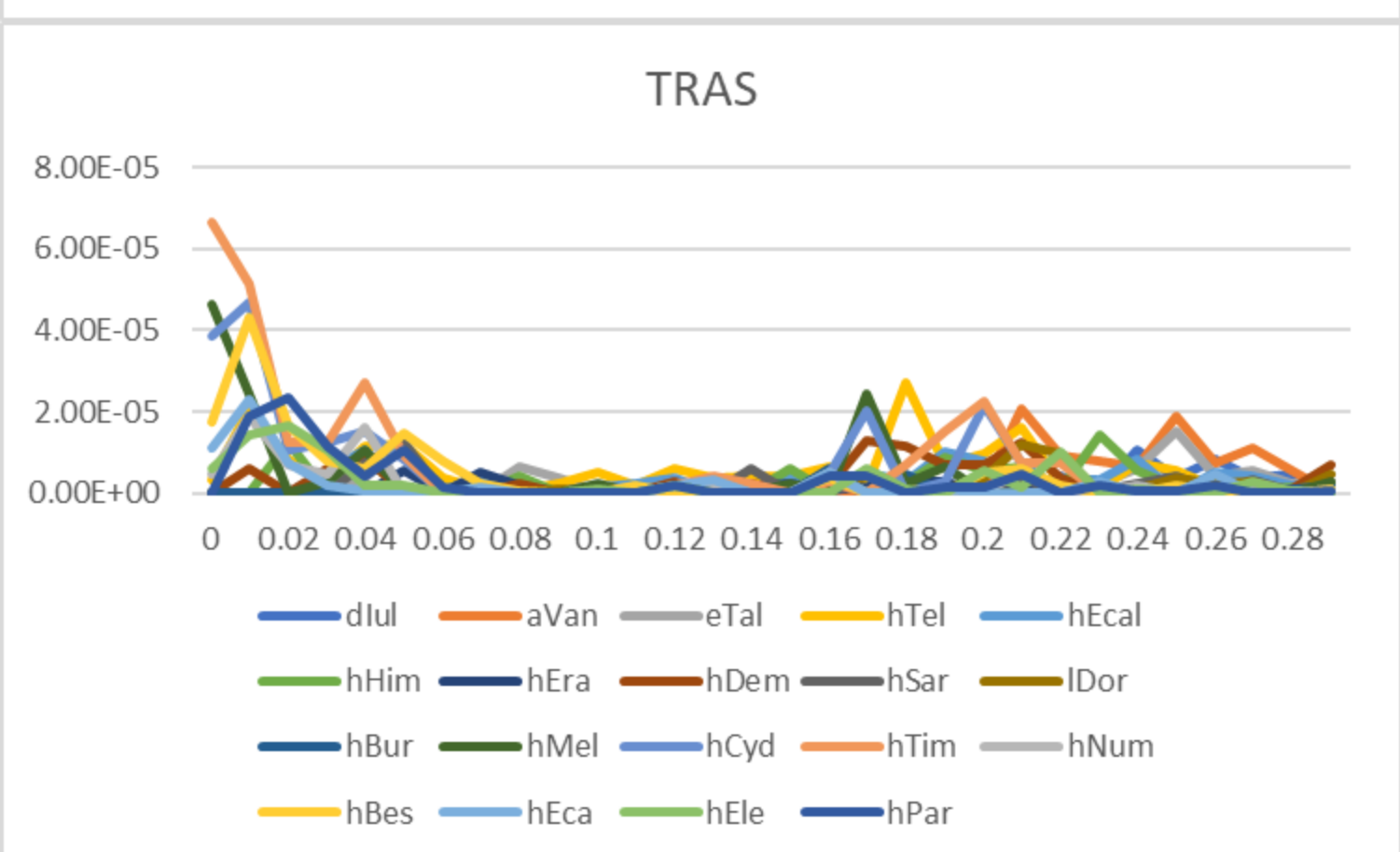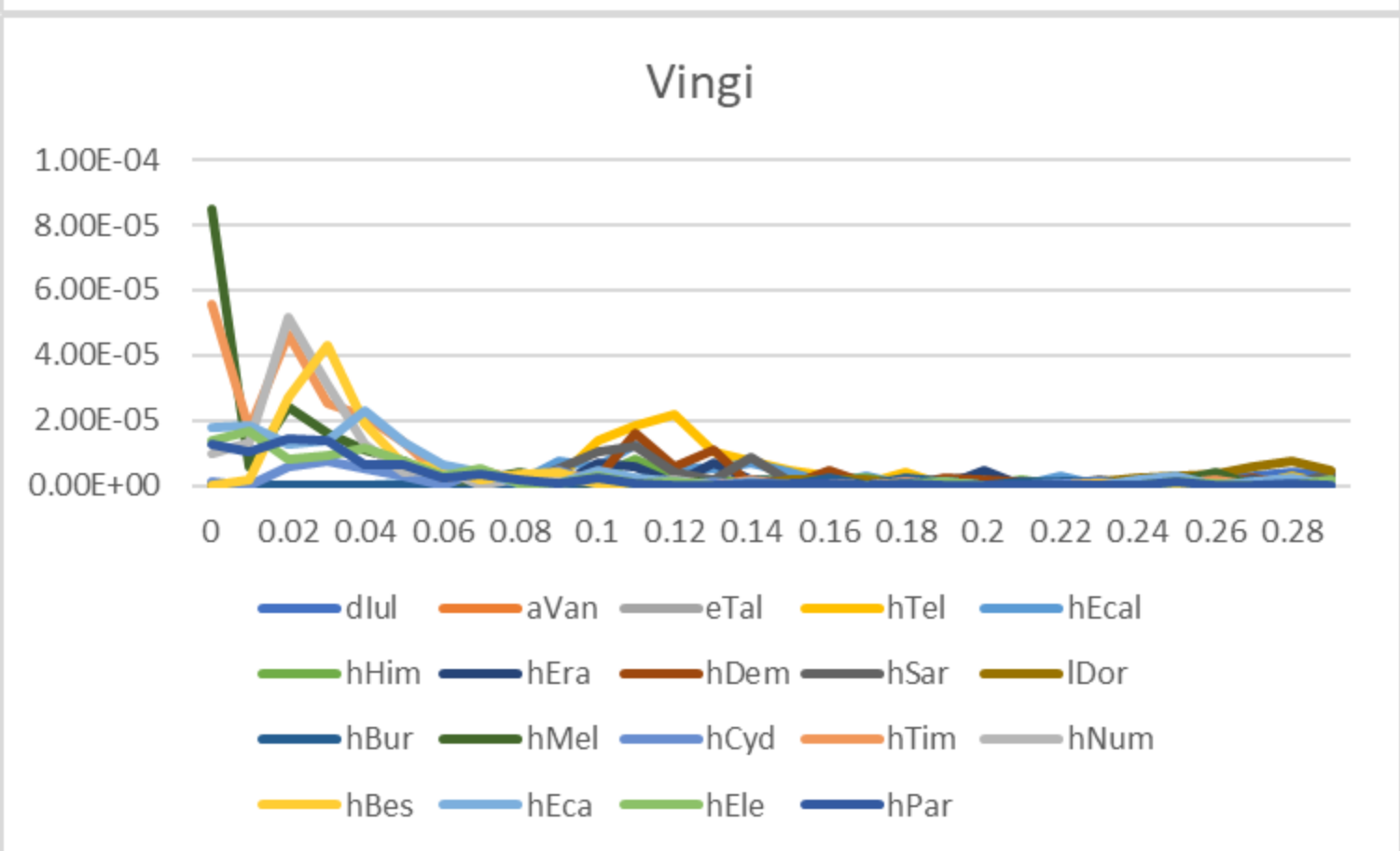

Supplement: evz125_Supplementary_Data [file evz125_supplementary_data.zip › Supplemental_Figure_12_-_LINE_plots.pdf]

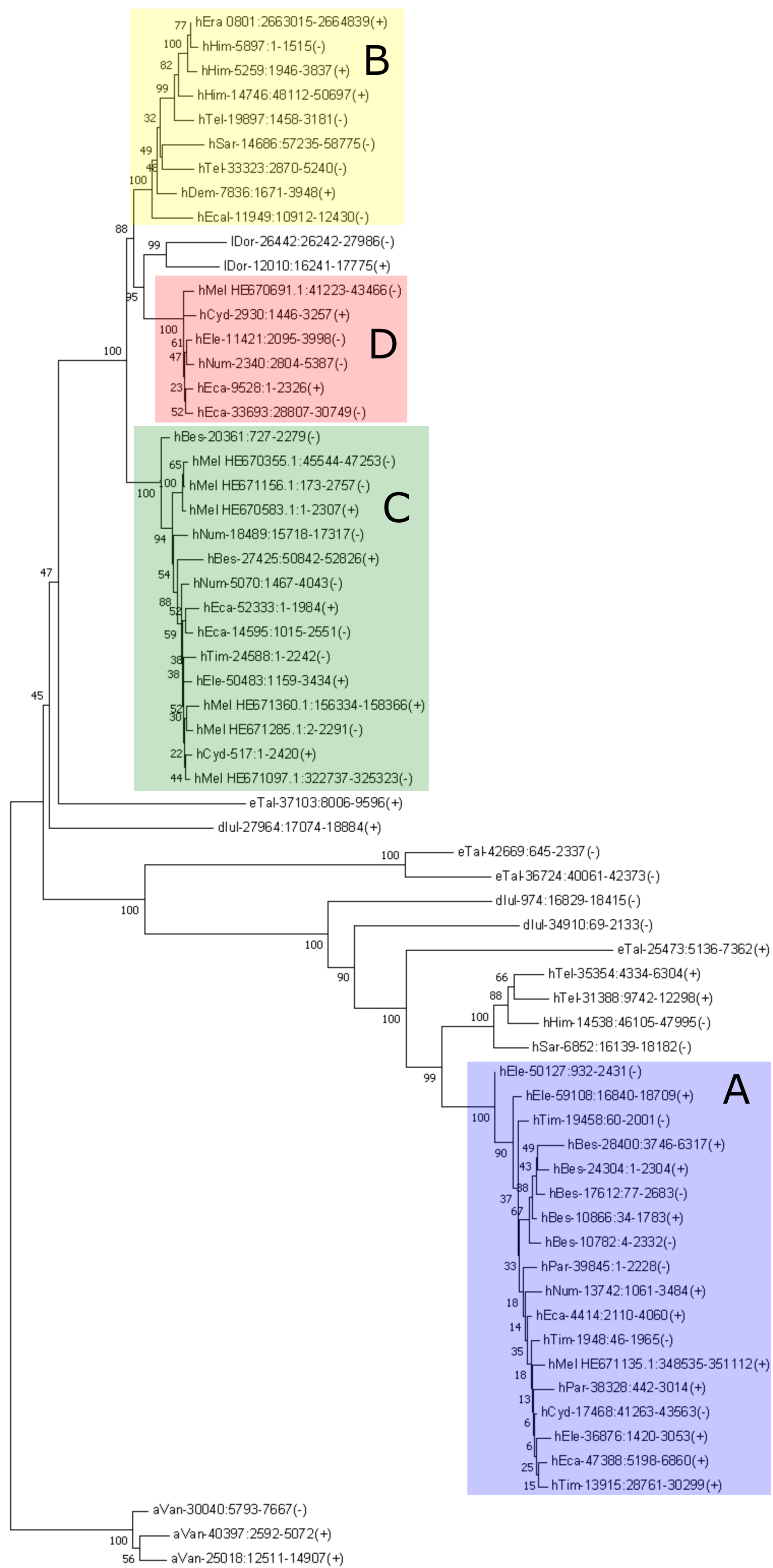

Supplement: evz125_Supplementary_Data [file evz125_supplementary_data.zip › Supplemental_Figure_13_-_RTE-4.pdf]

erato clade PSMC

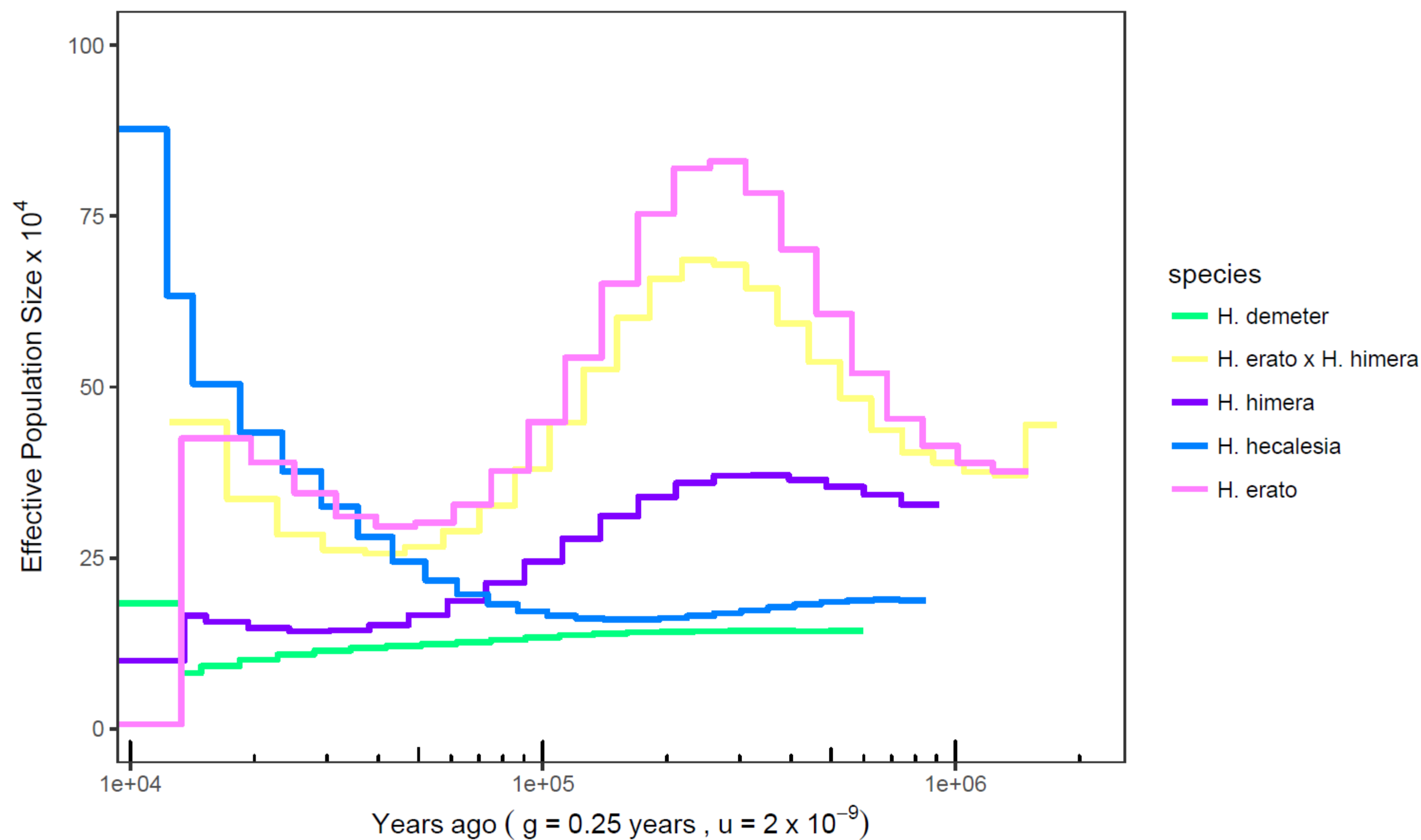

melpomene-silvaniform clade PSMC

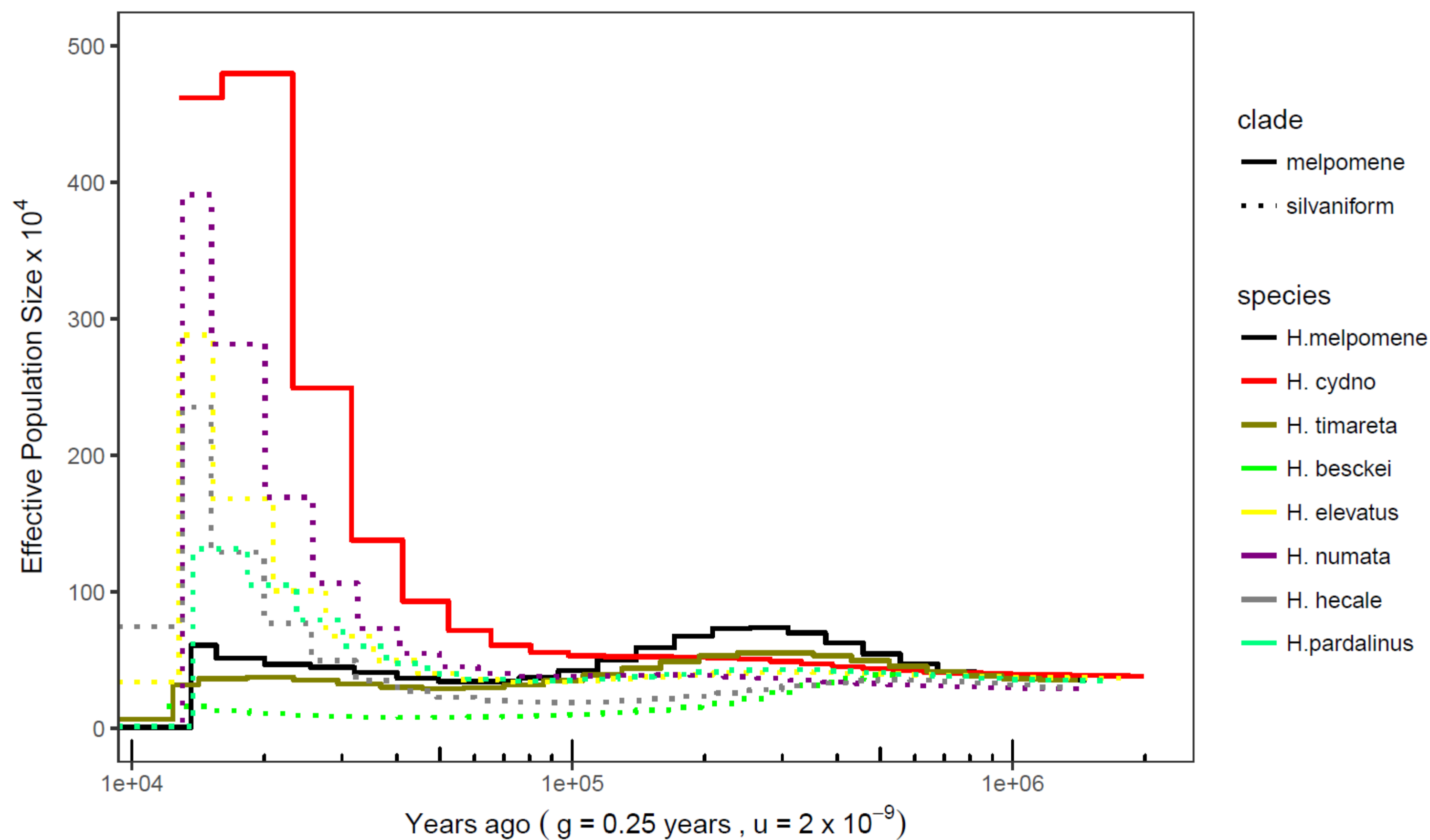

Supplement: evz125_Supplementary_Data [file evz125_supplementary_data.zip › Supplemental_Figure_14_-_psmc.pdf]

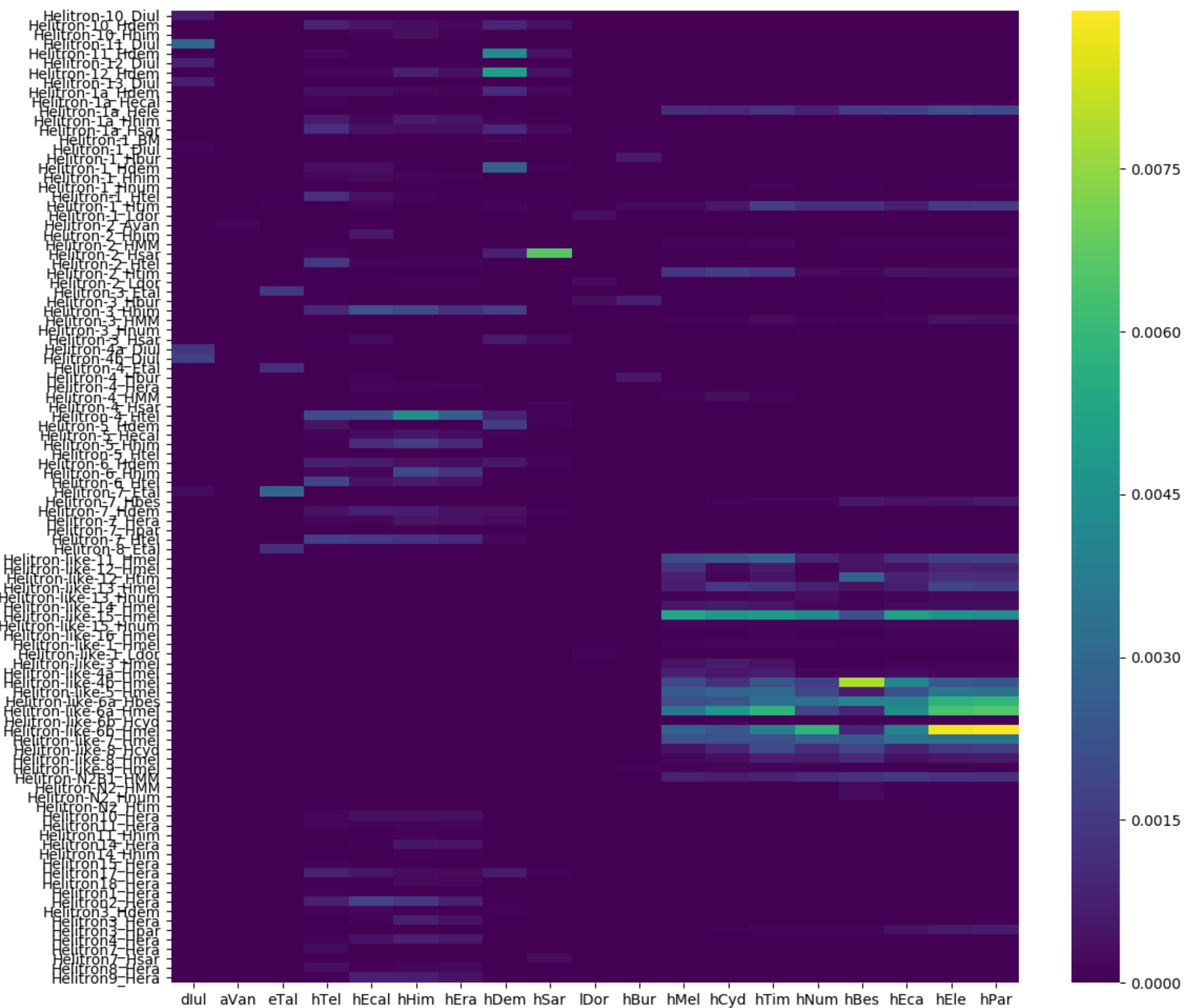

Supplement: evz125_Supplementary_Data [file evz125_supplementary_data.zip › Supplemental_Figure_2_-_RCpropheatmap.pdf]

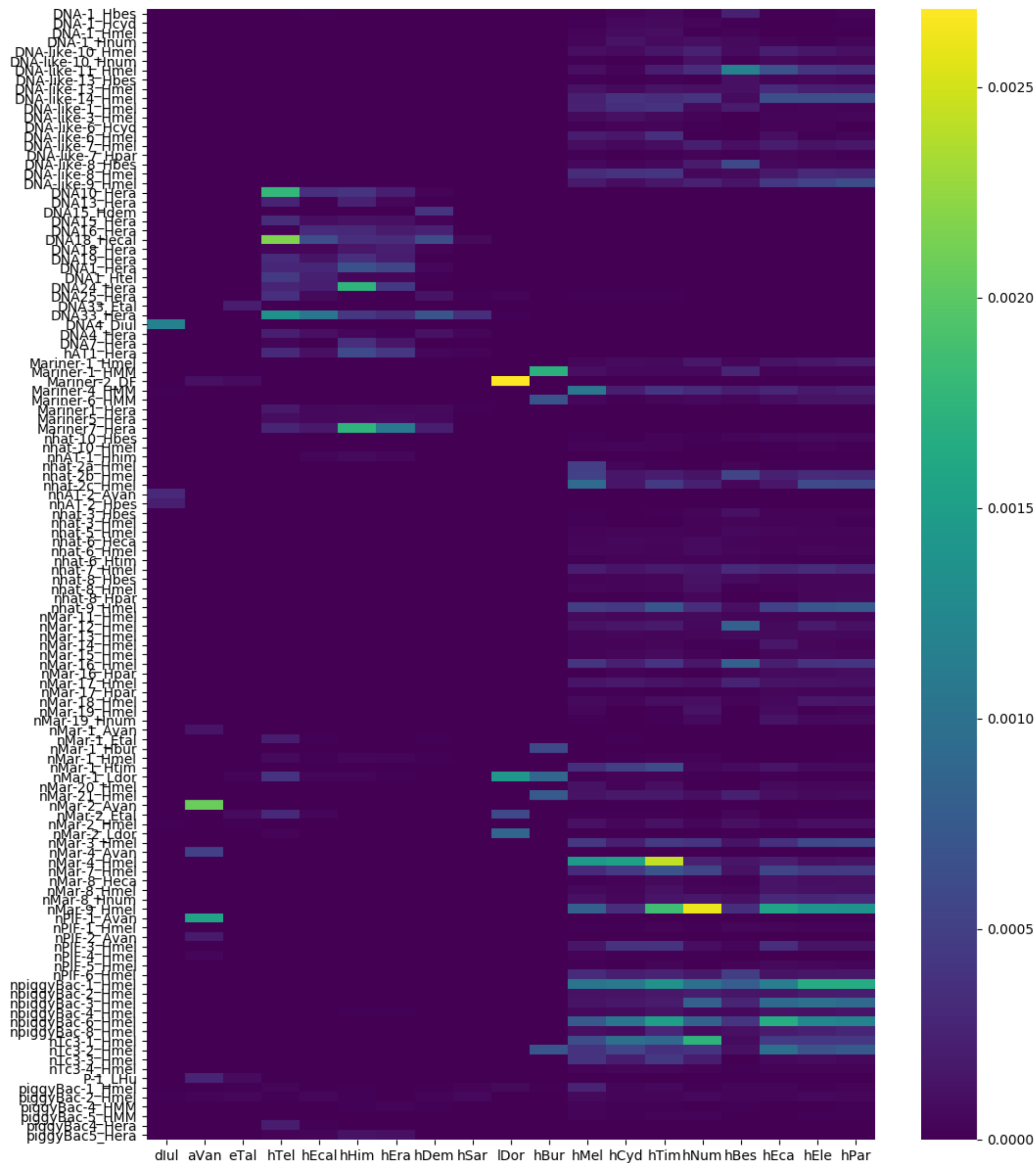

Supplement: evz125_Supplementary_Data [file evz125_supplementary_data.zip › Supplemental_Figure_3_-_DNApropheatmap.pdf]

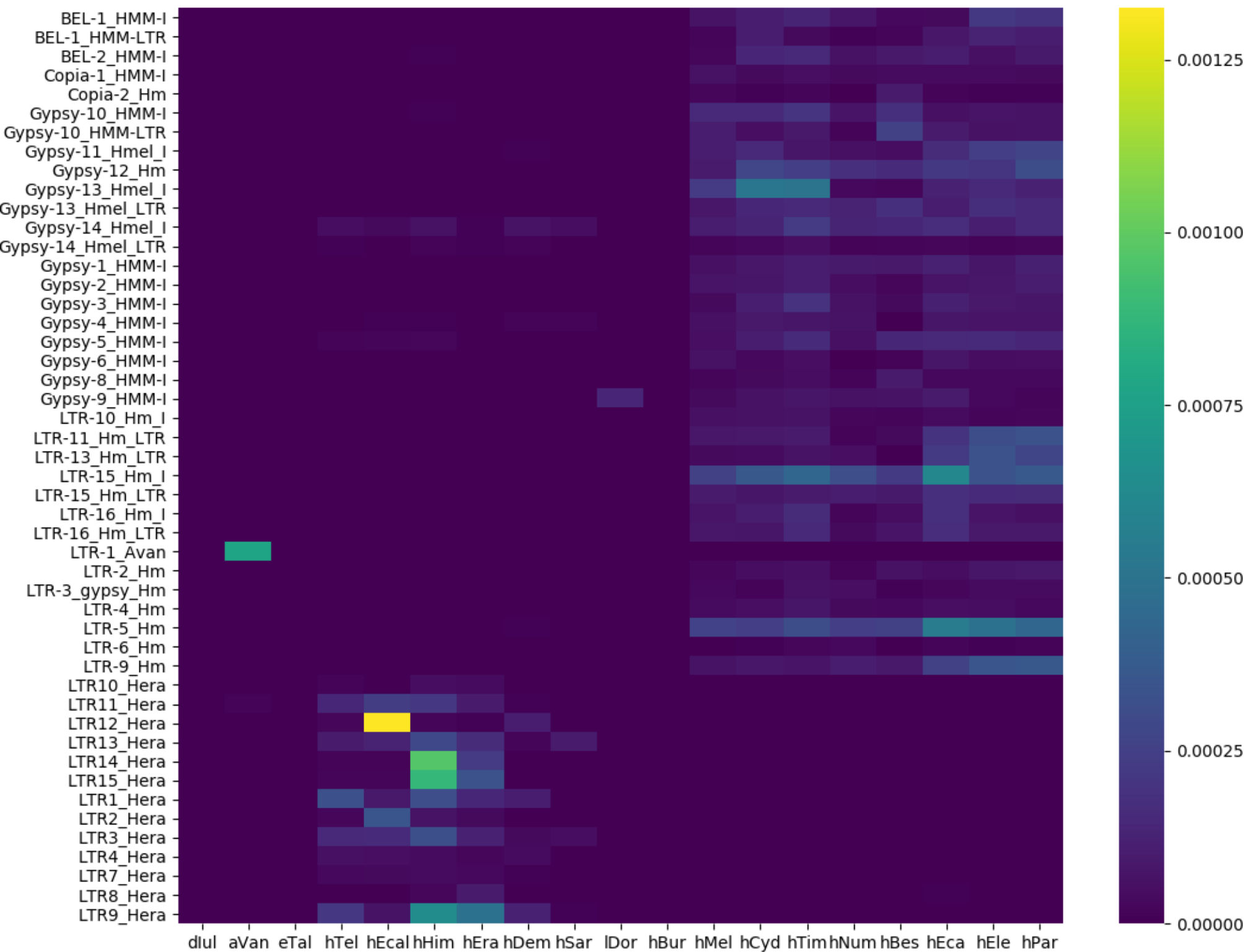

Supplement: evz125_Supplementary_Data [file evz125_supplementary_data.zip › Supplemental_Figure_4_-_LTRpropheatmap.pdf]

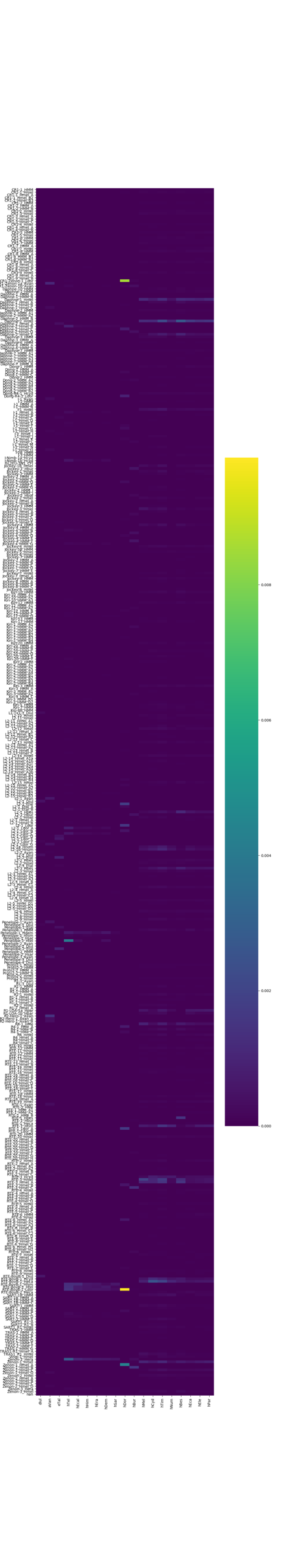

Supplement: evz125_Supplementary_Data [file evz125_supplementary_data.zip › Supplemental_Figure_5_-_LINEpropheatmap.pdf]

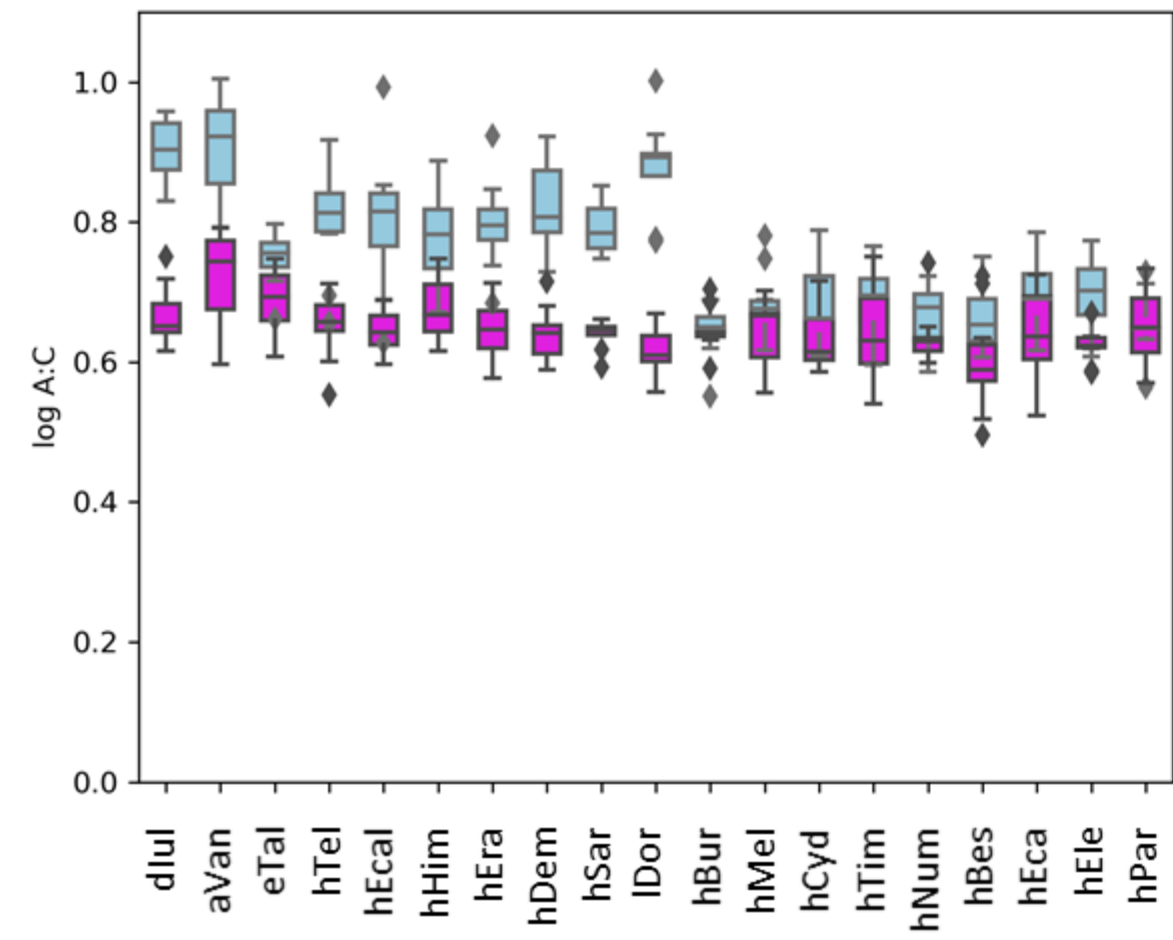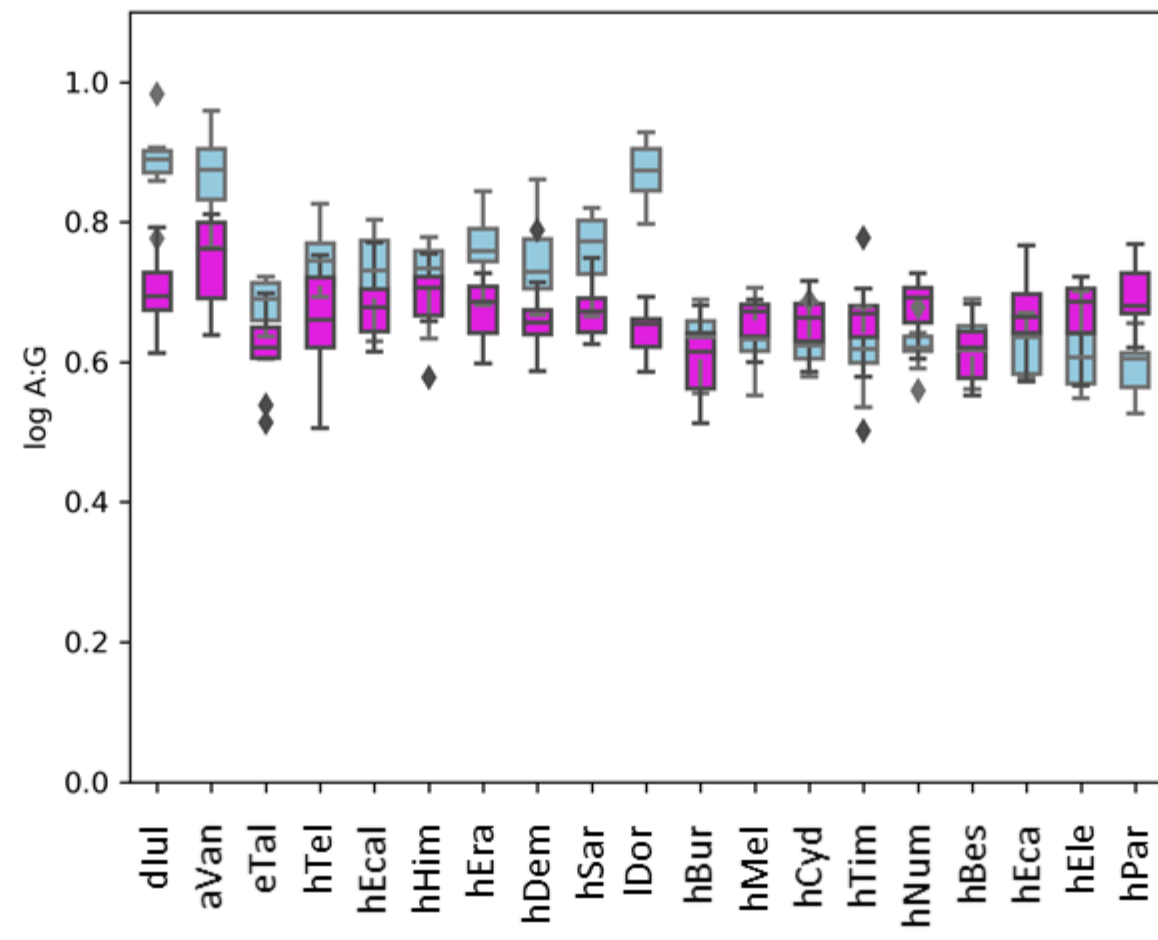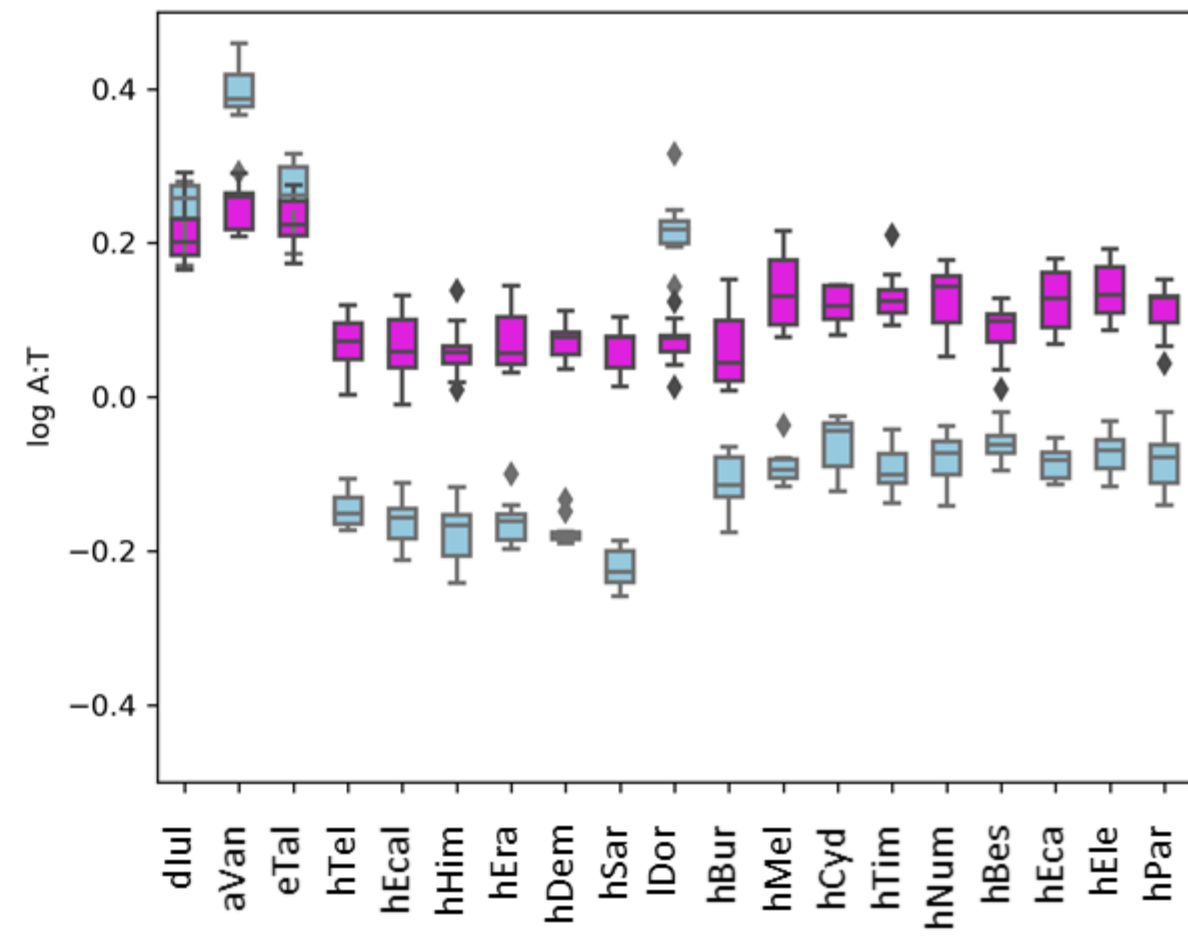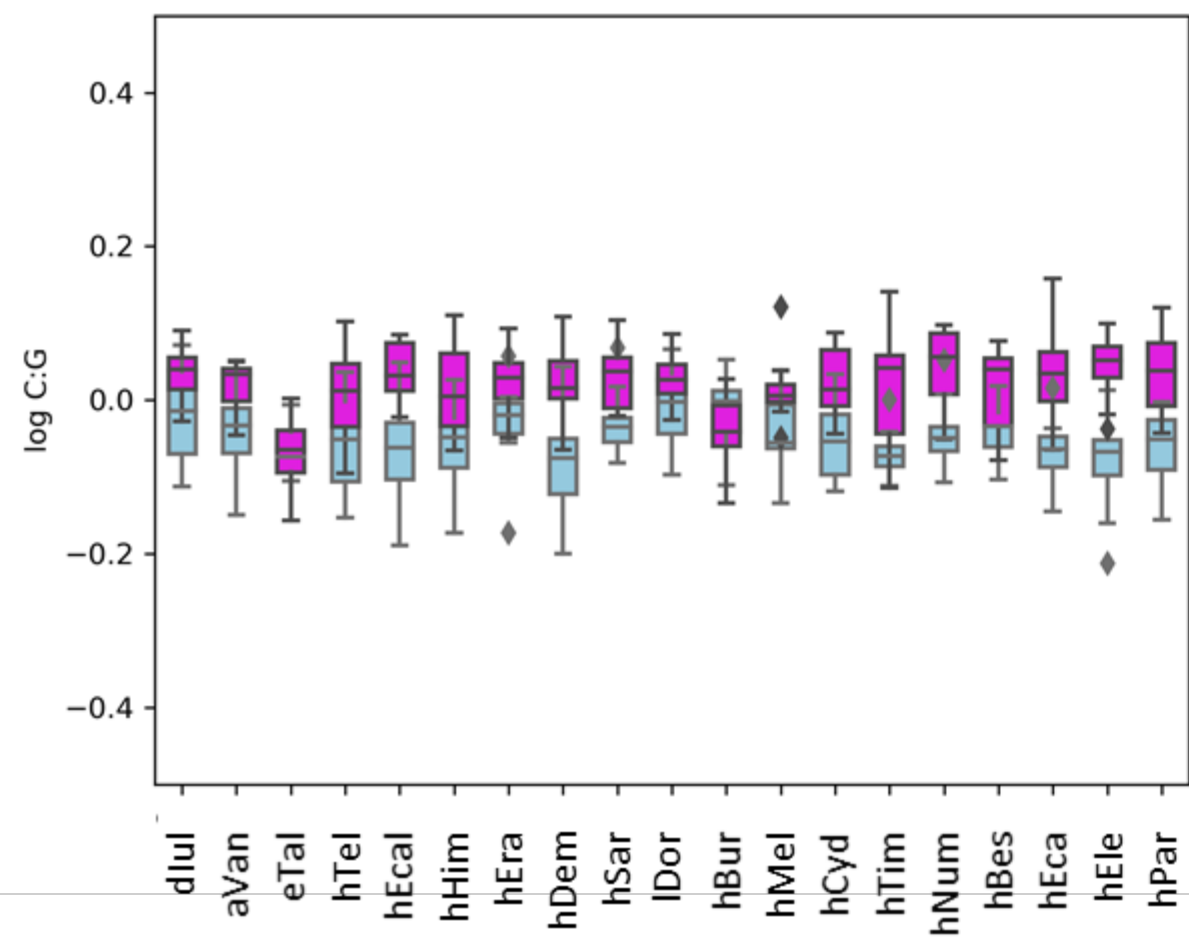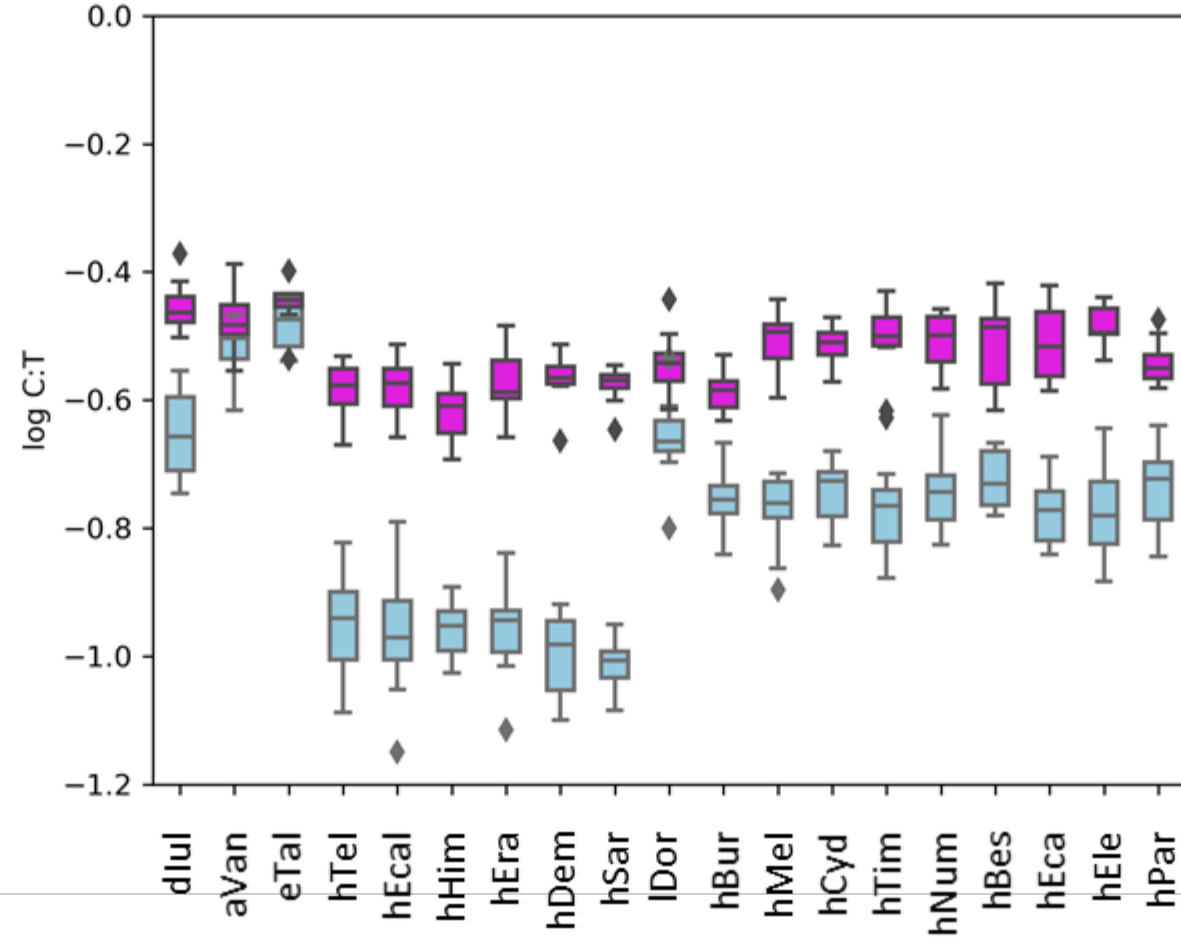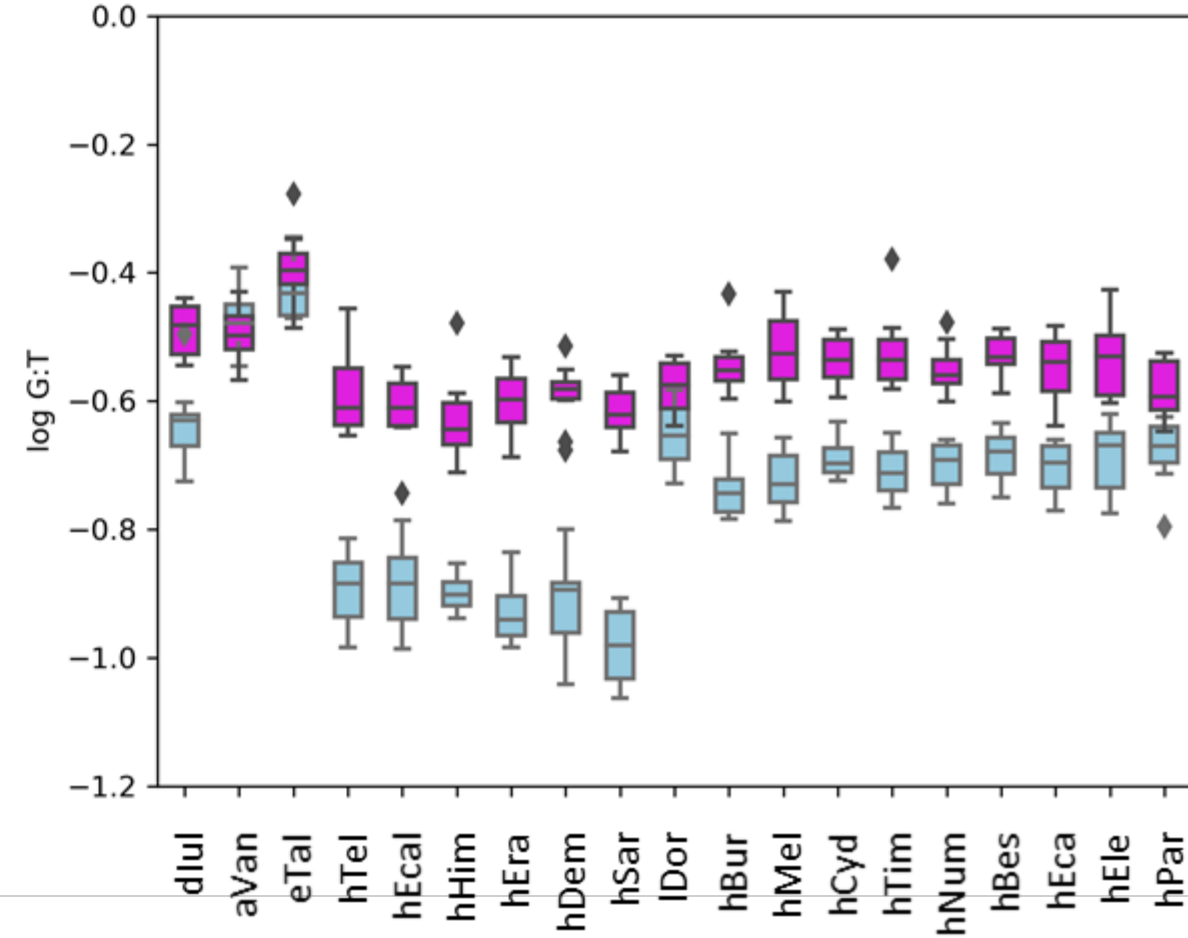

Supplement: evz125_Supplementary_Data [file evz125_supplementary_data.zip › Supplemental_Figure_8_-_Boxplots.pdf]

Metulj  
ZenoSINE

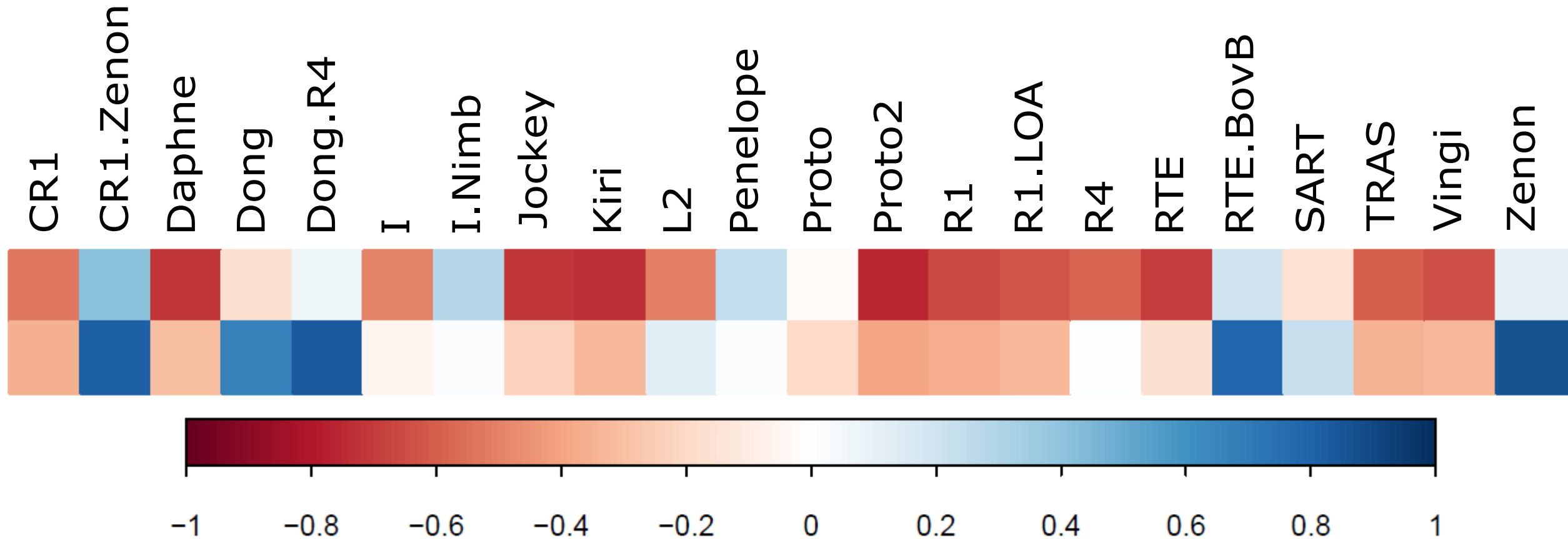

Supplement: evz125_Supplementary_Data [file evz125_supplementary_data.zip › Supplemental_Figure_9_-_Correlation_heatmap.pdf]
