# Supplementary material for: Simultaneous TE Analysis of 19 Heliconiine Butterflies Yields Novel Insights into Rapid TE-Based Genome Diversification and Multiple SINE Births and Deaths
Source: Genome Biol Evol. 2019 Jun 19;11(8):2162–77. doi: 10.1093/gbe/evz125 (PMC6685494; doi:10.1093/gbe/evz125)
Supplement: evz125_Supplementary_Data [file Supplemental_Figure_1_-_PCAs.pdf › Supplemental_Figure_1_-_PCAs.pdf]

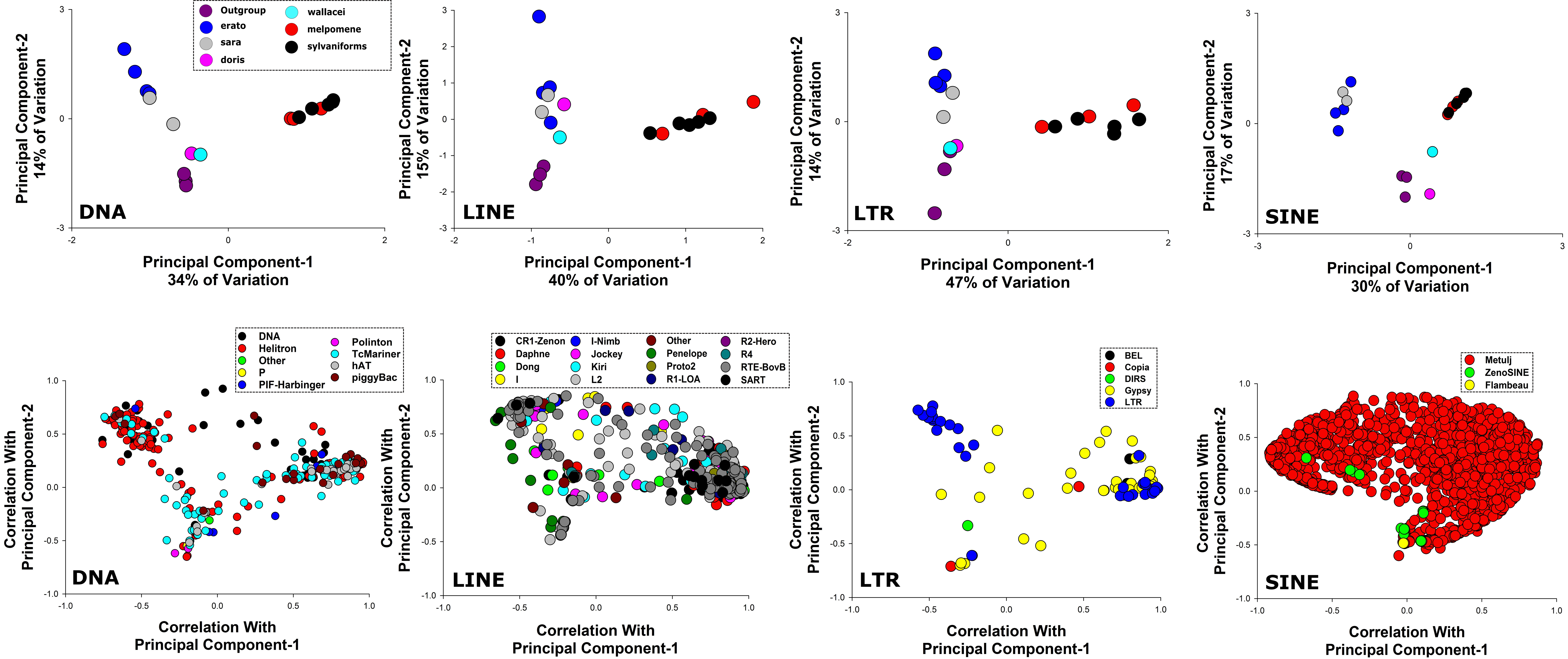

Outgroup
erato
sara
doris
wallacei
melpomene
sylvaniforms
Principal Component-2
14% of Variation
Principal Component-1
34% of Variation
DNA
Principal Compnent-2
15% of Variation
Principal Component-1
40% of Variation
LINE
Principal Component-2
14% of Variation
Principal Component-1
47% of Variation
LTR
Principal Component-2
17% of Variation
Principal Component-1
30% of Variation
SINE
DNA
Helitron
Other
P
PIF-Harbinger
Polinton
TcMariner
hAT
piggyBac
Correlation With
Principal Component-2
Correlation With
Principal Component-1
CR1-Zenon
Daphne
Dong
I
I-Nimb
Jockey
Kiri
L2
Other
Penelope
Proto2
R1-LOA
R2-Hero
R4
RTE-BovB
SART
BEL
Copia
DIRS
Gypsy
LTR
Metulj
ZenoSINE
Flambeau
