# Supplementary material for: Simultaneous TE Analysis of 19 Heliconiine Butterflies Yields Novel Insights into Rapid TE-Based Genome Diversification and Multiple SINE Births and Deaths
Source: Genome Biol Evol. 2019 Jun 19;11(8):2162–77. doi: 10.1093/gbe/evz125 (PMC6685494; doi:10.1093/gbe/evz125)

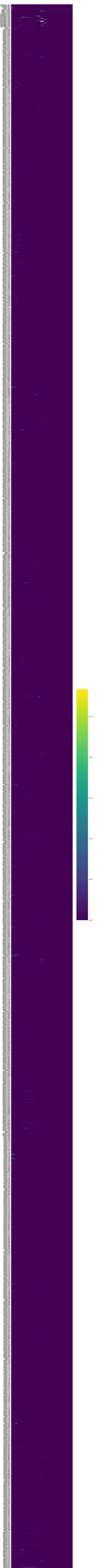

Supplement: evz125_Supplementary_Data [file Supplemental_Figure_6_-_SINEpropheatmap.pdf › Supplemental_Figure_6_-_SINEpropheatmap.pdf]
